# The Replisome-Coupled E3 Ubiquitin Ligase Rtt101^Mms22^ Counteracts Mrc1 Function to Tolerate Genotoxic Stress

**DOI:** 10.1371/journal.pgen.1005843

**Published:** 2016-02-05

**Authors:** Raymond Buser, Vanessa Kellner, Andre Melnik, Caroline Wilson-Zbinden, René Schellhaas, Lisa Kastner, Wojciech Piwko, Martina Dees, Paola Picotti, Marija Maric, Karim Labib, Brian Luke, Matthias Peter

**Affiliations:** 1 Institute of Biochemistry, Department of Biology, ETH Zurich, Zürich, Switzerland; 2 Zentrum für Molekulare Biologie der Universität Heidelberg (ZMBH), Heidelberg, Germany; 3 MRC Protein Phosphorylation and Ubiquitylation Unit, College of Life Sciences, University of Dundee, Dundee, Scotland, United Kingdom; California Institute of Technology, UNITED STATES

## Abstract

Faithful DNA replication and repair requires the activity of cullin 4-based E3 ubiquitin ligases (CRL4), but the underlying mechanisms remain poorly understood. The budding yeast Cul4 homologue, Rtt101, in complex with the linker Mms1 and the putative substrate adaptor Mms22 promotes progression of replication forks through damaged DNA. Here we characterized the interactome of Mms22 and found that the Rtt101^Mms22^ ligase associates with the replisome progression complex during S-phase via the amino-terminal WD40 domain of Ctf4. Moreover, genetic screening for suppressors of the genotoxic sensitivity of *rtt101Δ* cells identified a cluster of replication proteins, among them a component of the fork protection complex, Mrc1. In contrast to *rtt101Δ* and *mms22Δ* cells, *mrc1Δ rtt101Δ* and *mrc1Δ* *mms22Δ* double mutants complete DNA replication upon replication stress by facilitating the repair/restart of stalled replication forks using a Rad52-dependent mechanism. Our results suggest that the Rtt101^Mms22^ E3 ligase does not induce Mrc1 degradation, but specifically counteracts Mrc1’s replicative function, possibly by modulating its interaction with the CMG (Cdc45-MCM-GINS) complex at stalled forks.

## Author Summary

Post-translational protein modifications, such as ubiquitylation, are essential for cells to respond to environmental cues. In order to understand how eukaryotes cope with DNA damage, we have investigated a conserved E3 ubiquitin ligase complex required for the resistance to carcinogenic chemicals. This complex, composed of Rtt101, Mms1 and Mms22 in budding yeast, plays a critical role in regulating the fate of stalled DNA replication. Here, we found that the Rtt101^Mms22^ E3 ubiquitin ligase complex interacts with the replisome during S-phase, and orchestrates the repair/restart of DNA synthesis after stalling by activating a Rad52-dependent homologous recombination pathway. Our findings indicate that Rtt101^Mms22^ specifically counteracts the replicative activity of Mrc1, a subunit of the fork protection complex, possibly by modulating its interaction with the CMG (Cdc45-MCM-GINS) helicase complex upon fork stalling. Altogether, our study unravels a functional protein cluster that is essential to understand how eukaryotic cells cope with DNA damage during replication and, thus deepens our knowledge of the biology that underlies carcinogenesis.

## Introduction

DNA replication is a process through which cells duplicate their entire genome prior to cell division. To achieve accurate replication, eukaryotes have evolved intricate surveillance systems that allow fine-tuning of the replication machinery. In order to continually provide the replicative polymerase with a single stranded DNA template, replisomes must adapt to chromatin heterogeneities such as aberrant DNA structures, condensed chromatids, transcriptional obstacles and DNA-protein barriers [1]. In *Saccharomyces cerevisiae*, this adaptation is regulated by proteins such as Mrc1, Tof1, Csm3 and Ctf4, which assemble around the CMG (Cdc45-MCM-GINS) DNA helicase at replication forks. These components form the ‘Replisome Progression Complex’ (RPC), a replisome sub-assembly that exists exclusively at replication forks [2]. The RPC functions in coupling DNA polymerases to the CMG helicase [3,4], and in regulating fork progression [5–9]. Moreover, these replisome components also limit mutagenic frequency and prevent unscheduled homologous recombination (HR) events at stalled forks [10–12]. Mrc1 possesses two polymerase epsilon (Pol ε) binding sites [13] as well as an Mcm6 interaction motif [14], and is required for checkpoint activation in response to replication stress [15]. Tof1 and Csm3 help to link Mrc1 to fork components [16,17] but also have distinct functions not shared with Mrc1 [9]. Conversely, the replication fork progression defect is enhanced in *mrc1Δ* compared to *tof1Δ* and *csm3Δ* mutants [1], implying that Mrc1 also promotes replication functions independent of Tof1 and Csm3. Ctf4, the yeast homologue of human AND1, bridges the interaction of the primase, DNA polymerase-α, to the CMG helicase [3,5,18]. Although the coupling of the CMG to leading and lagging strand DNA polymerases preserves genome integrity during unperturbed DNA replication, this mechanism is partially disrupted when forks encounter replication stress. Indeed, significant stretches of ssDNA generated by uncoupling can promote HR-mediated replication re-start via either a template switch or break-induced replication (BIR) [19].

Growing evidence implicates Cullin-RING containing E3 ligases (CRL’s) in regulating DNA replication and repair [20]. For example, Cdc53/Cul1 in a complex with the F-box adaptor protein Dia2 (SCF^Dia2^) promotes the ubiquitylation of the Mcm7 subunit of the CMG helicase, which triggers Cdc48/p97-dependent disassembly of CMG at the end of DNA replication [21]. In addition, SCF^Dia2^ has been reported to antagonize Mrc1 upon replication stress, possibly by inducing degradation of phosphorylated Mrc1 to recover from checkpoint arrest following repair [22–24]. Cullin4 (CRL4)-based E3 ubiquitin ligases regulate DNA replication and repair both in yeast and mammalian cells, in part by controlling histone dynamics at active replication forks [25]. Rtt101, the budding yeast analogue of human Cul4 [26], has been reported to target Spt16, a subunit of the FACT complex that reorganizes nucleosomes during DNA replication [27]. Moreover, Rtt101 promotes replication fork progression through DNA lesions and natural pause sites [28]. This function is dependent on *MMS1* and *MMS22*, which encode a DDB1-like linker protein and a putative substrate specific adaptor, respectively [26]. However, the underlying mechanisms and function of the Rtt101-Mms1-Mms22 complex (termed Rtt101^Mms22^) remain largely elusive. Recent results suggest that the sensitivity of *mms1Δ*, *mms22Δ* and *rtt101Δ* cells to the DNA-damaging drugs CPT and MMS could be rescued by further deleting *MRC1* [18,29], possibly by de-regulating late firing origins [29].

Here we show that the Rtt101^Mms22^ E3 ubiquitin ligase genetically and biochemically interacts with components of the replication fork. We found that the WD40 domain of Ctf4, a protein required for coupling the CMG complex to the replicative polymerases, recruits Mms22 to active forks during S-phase. Moreover, Mms22 physically associates with Mrc1 and deletion of the *MRC1* gene suppresses the defects of *rtt101Δ*, *mms1Δ* and *mms22Δ* cells, including genotoxic sensitivity, prolonged checkpoint activation and reduced HR rates. Importantly, our results suggest that de-repression of late replication origins is not sufficient to bypass the need of Rtt101^Mms22^ E3-ligase activity. Instead, *MRC1* deletion promotes HR-mediated repair of replication forks that have paused in response to replication stress. Based on genetic and biochemical data we propose that the Rtt101^Mms22^ complex specifically counteracts the replicative, and not the checkpoint, function of Mrc1, possibly by modulating its interaction with the CMG helicase complex upon fork stalling.

## Results

### *RTT101*, *MMS1* and *MMS22* interact genetically with proteins involved in replication

**Fig 1:**
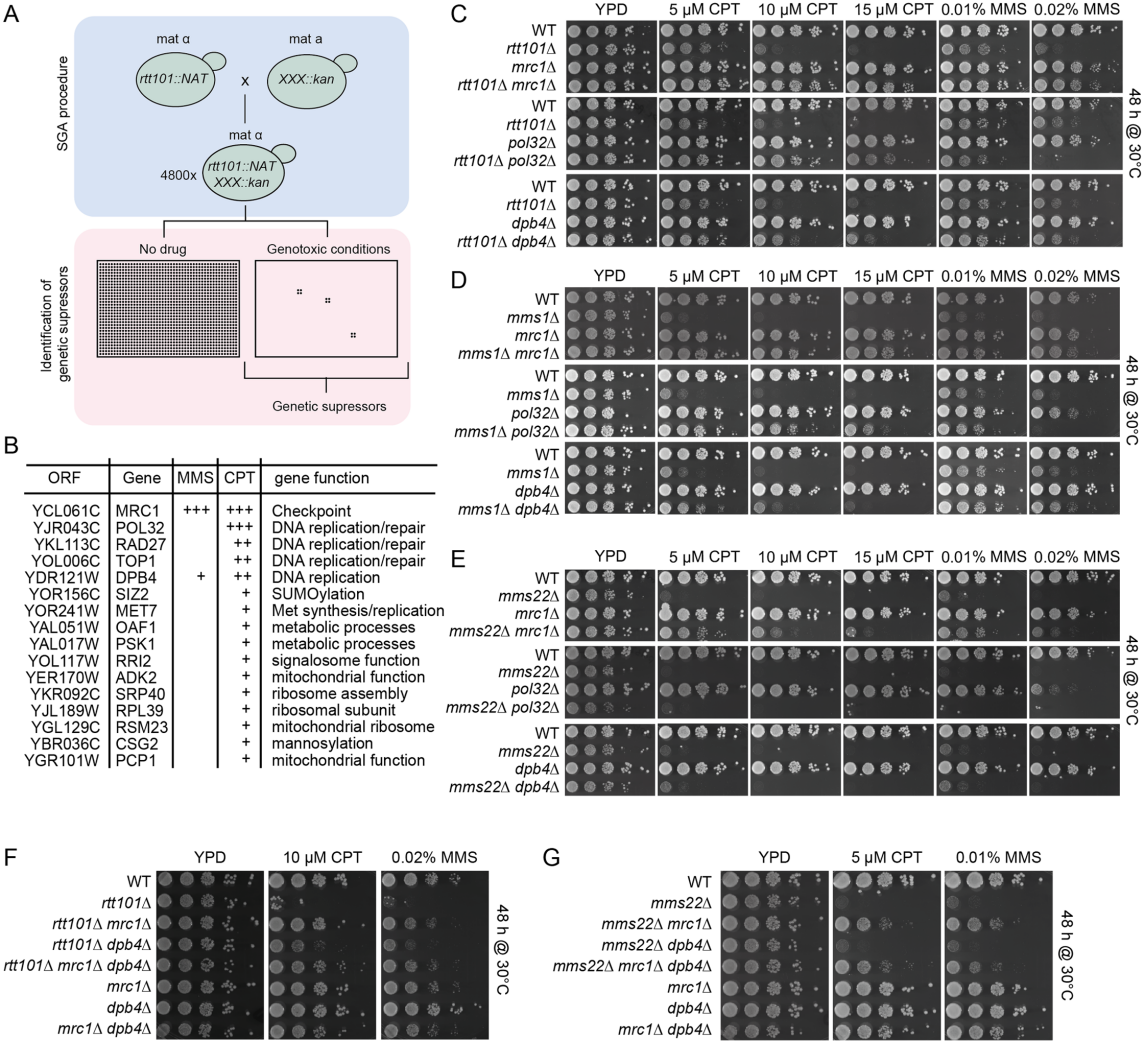
The Rtt101-Mms1-Mms22 E3 ubiquitin ligase genetically interacts with genes involved in DNA replication. (A) and (B) Schematic representation of the synthetic genetic array (SGA) screening procedure (A), and table summarizing cellular functions of the main hits (B). Genotoxic conditions included MMS (0.01%) and CPT (5 μM) and colony size was qualitatively scored after 72 hours at 30°C. (+) weak suppression, (++) medium suppression, (+++) strong suppression. See Materials and Methods for a detailed description of the screening procedure and S1 Fig for an example of the raw data. (C) *RTT101* genetically interacts with the replication genes *MRC1*, *POL32* and *DPB4*. Serial dilution of wild-type (WT) or *rtt101Δ*, *mrc1Δ*, *rtt101Δ* *mrc1Δ*, *pol32Δ*, *rtt101Δ* *pol32Δ*, *dpb4Δ* and *rtt101Δ* *dpb4Δ* mutants were assayed on normal growth media (YPD), and media containing 5 μM, 10 μM and 15 μM CPT or 0.01% and 0.02% MMS. Cells were imaged after 48 hours of incubation at 30°C.(D) *MMS1* phenocopies the genetic interactions of *RTT101* with *MRC1*, *POL32* and *DPB4*. Serial dilution of wild-type (WT) or *mms1Δ*, *mrc1Δ*, *mms1Δ* *mrc1Δ*, *pol32Δ*, *mms1Δ* *pol32Δ*, *dpb4Δ* and *mms1Δ* *dpb4Δ* mutants were assayed on normal growth media (YPD), and media containing 5 μM, 10 μM and 15 μM CPT or 0.01% and 0.02% MMS. Cells were imaged after 48 hours of incubation at 30°C. (E) *MMS22* phenocopies the genetic interactions of *RTT101* and *MMS1* with *MRC1* and *DPB4*, but not *POL32*. Serial dilution of wild-type (WT) or *mms22Δ*, *mrc1Δ*, *mms22Δ* *mrc1Δ*, *pol32Δ*, *mms22Δ* *pol32Δ*, *dpb4Δ* and *mms22Δ* *dpb4Δ* mutants were assayed on normal growth media (YPD), and media containing 5 μM, 10 μM and 15 μM CPT or 0.01% and 0.02% MMS. Cells were imaged after 48 hours of incubation at 30°C. (F) and (G). *mrc1Δ* and *dpb4Δ* rescue *rtt101Δ* and *mms22Δ* in an epistatic manner. Cells were spotted on the indicated media and imaged as described above in (C, D and E).

To elucidate the role of the Rtt101^Mms22^ E3 ubiquitin ligase in DNA replication, we employed an automated SGA approach [30] to screen for genes that, when deleted, would suppress the growth defects of *rtt101Δ* cells exposed to either the alkylating agent methyl methanesulfonate (MMS) or the topoisomerase 1 (Top1) poison camptothecin (CPT) [31] (Figs 1A and S1). The SGA screen was performed in duplicate and only suppressors that appeared in both screens were considered for further analysis. The combined results for MMS and CPT conditions initially identified 63 suppressor genes that were scored by visual inspection as either strong, medium or weak (S1 Table). Only the strong and medium hits were validated by crossing the single deletion mutants to an independent *rtt101Δ* strain, in which double mutants were derived by manual tetrad dissection. Cells were then spotted in biological duplicate on MMS and CPT containing media as depicted in Fig 1C–1E. Using this workflow, we confirmed a list of 16 genes that when deleted improved the growth of *rtt101Δ* cells (Fig 1B). Among the most potent suppressors are genes directly involved in DNA replication, including *MRC1*, *POL32*, *RAD27*, *TOP1*, *SIZ2* and *DPB4*. Additional suppressor mutations implicated in other biological processes were also confirmed (Fig 1B), but were not further characterized in this study. Some suppressors such as the lagging strand polymerase subunit Pol32 only restored growth on CPT containing media (Fig 1B–1D), indicating that the screening approach could isolate functional protein sub-clusters. The slow growth phenotype of other Rtt101-based E3 ubiquitin ligase component mutants, including those lacking the linker protein Mms1 (Fig 1D) as well as mutants deleted for the putative substrate specific adaptor Mms22 (Fig 1E), were rescued by deleting the identical replication gene cluster that suppressed the *rtt101Δ* cell phenotype. These data indicate that Rtt101 likely acts as a fully assembled E3 ubiquitin ligase in a process associated with replication stress. Indeed, deletion of *MRC1* suppressed the growth defects of cells expressing the neddylation-deficient Rtt101-K791R mutant [32], implying that loss of *MRC1* suppresses the phenotypes correlated with inactivation of Rtt101^Mms22^ E3 ligase activity (S2 Fig). In contrast, deletion of *MRC1* did not rescue the MMS sensitivity of *ubc13Δ* cells defective for PCNA polyubiquitylation (S3 Fig), indicating that the genetic suppression is specific to the loss of Rtt101^Mms22^ function and not other ubiquitylation-defective mutants involved in lesion bypass repair [33].

*MRC1* and *DPB4* are both linked to the putative leading strand polymerase, Pol ε, and deletion of these genes showed suppression on both MMS and CPT containing media, albeit to varying extents. Furthermore, when the deletions of *DPB4* and *MRC1* were combined in the absence of either *RTT101* or *MMS22*, we observed that the genetic rescue was not additive (Fig 1F and 1G). Unlike in *rtt101Δ* or *mms1Δ* cells, the deletion of *POL32* did not rescue the CPT sensitivity of *mms22Δ* cells (Fig 1E), supporting the notion that Mms22 has additional functions independent of the E3 ligase complex [34]. Taken together, these data demonstrate that deletion of replication genes such as *MRC1* and *DPB4* can alleviate the growth defects associated with impaired Rtt101^Mms22^ E3 ubiquitin ligase activity in response to multiple genotoxic agents.

### The Rtt101^Mms22^ complex associates with replisomes during S-phase

**Fig 2:**
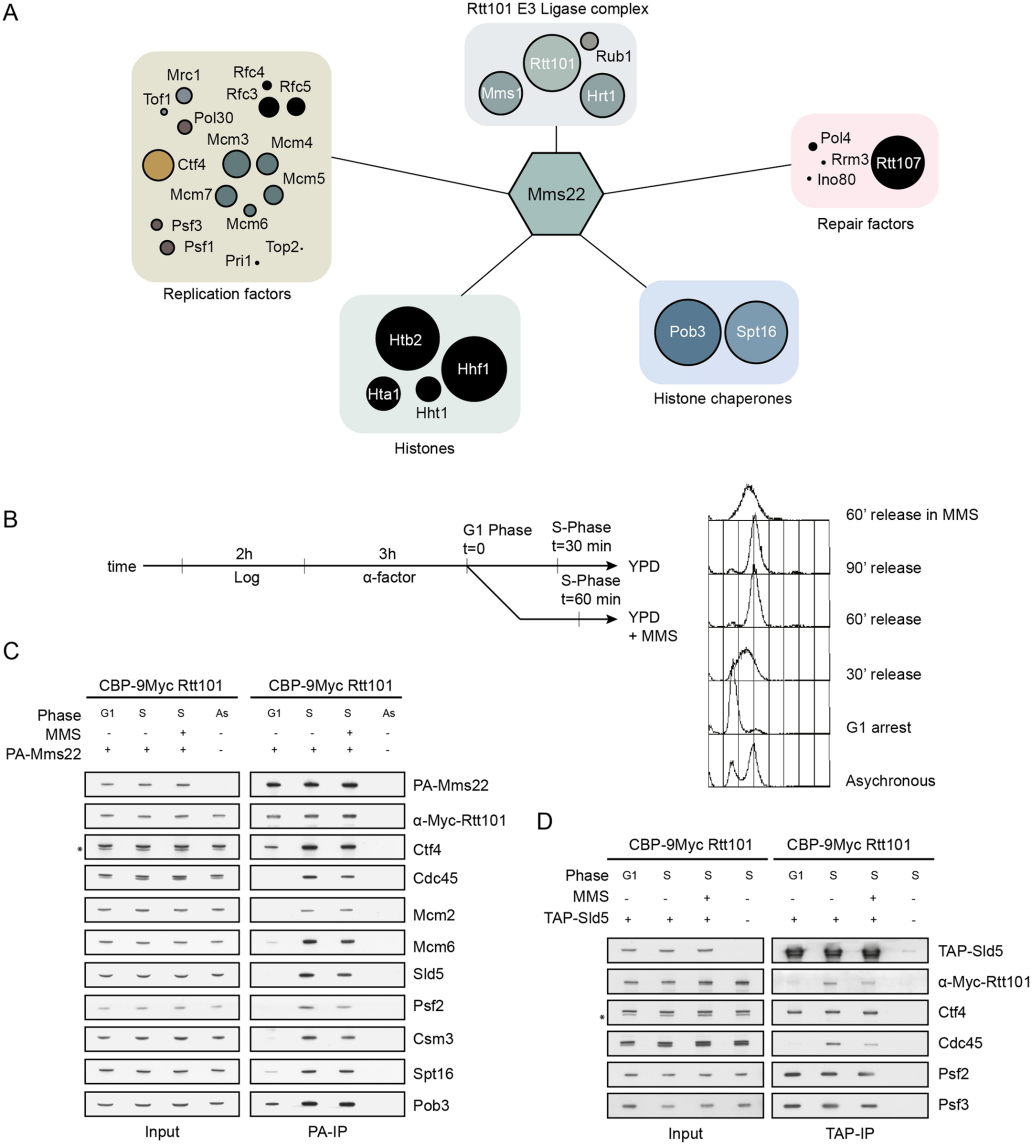
The substrate-specific adaptor Mms22 physically interacts with replisome components during S-phase. (A) The S-phase specific Mms22 interaction network identified by MS/MS. PA-tagged Mms22 was immunoprecipitated from cells synchronized in S-phase and associated proteins were identified by MS-analysis. The hits were grouped according to known functions and the ring diameter is proportional to the percent of coverage measured for the indicated proteins. (B) and (C) Mms22 physically interacts with components of the replisome during S-phase. Cells endogenously expressing CBP-9Myc-Rtt101 were transformed with PA-Mms22 and synchronized in S-phase by release from α-factor arrest as schematically outlined (B), and either treated or not with 0.03% MMS to induce fork stalling. Visualizing the DNA content by flow cytometry monitored cell synchronization. PA-Mms22 was immunoprecipitated from the indicated cell extracts and associated proteins detected by immunoblotting using specific antibodies (C). The asterisk (*) indicates an unspecific band. (D) S-phase specific interaction of Rtt101 with replisome components. Cells endogenously expressing CBP-9Myc-Rtt101 and TAP-Sld5 were synchronized and treated as in (C). TAP-Sld5 was purified from the indicated extracts and interacting proteins visualized by immunoblotting with specific antibodies.

The above genetic data suggests that the Rtt101^Mms22^ complex may directly interact with the replisome. Since specificity of a CRL complex is mainly conferred by the substrate adaptor [35], we immunoprecipitated Mms22 from S-phase synchronized cells and identified associated proteins by an unbiased mass-spectrometry method referred to as shotgun LC-MS/MS (Fig 2A). As expected, Mms22 co-purified with the E3 ligase subunits Rtt101, Mms1 and Hrt1, with the core histones Htb2, Hhf1, Hta1, and Hht1, as well as with the FACT complex (Spt16, Pob3), a nucleosome re-organizer that likely facilitates the interactions between DNA replication and transcription. These findings are consistent with previously published data showing that Rtt101-Mms1 associates with histone H3 [25] and ubiquitylates the Spt16 subunit of FACT [27]. We also detected a cluster of replication factors, including components of the GINS- (go-ichi-ni-san), the fork protection- and the MCM helicase complexes (Fig 2A, see also S2 Table for a complete list of Mms22-interacting proteins). These results strengthen the genetic interaction clusters that were found to suppress the growth defects of *rtt101Δ* cells, and strongly suggest a function of Rtt101^Mms22^ at replisomes during S-phase.

To better characterize the interaction of Mms22 with replisome components, we immunoprecipitated functional epitope-tagged versions of Mms22 (PA-Mms22) (Fig 2C) and the GINS complex (TAP-Sld5) (Fig 2D) from cells synchronized in G1 and S-phase with normal or genotoxic stress (0.03% MMS) growth conditions (Fig 2B). We observed that Mms22 and Rtt101 interact with all tested components of the replisome progression complex during S-phase (Fig 2C and 2D). Remarkably, Ctf4 and the FACT complex showed affinity for Mms22 in both G1 and S-phase, although both interactions were more prominent during S-phase. The induction of DNA damage through MMS treatment did not alter the observed interactions, suggesting that the Rtt101^Mms22^ E3 ligase is not specifically recruited to replisomes upon genotoxic insult, but rather constitutively associates with the active replication machinery.

### Ctf4 tethers the Rtt101^Mms22^ E3 ubiquitin ligase to active replisomes

**Fig 3:**
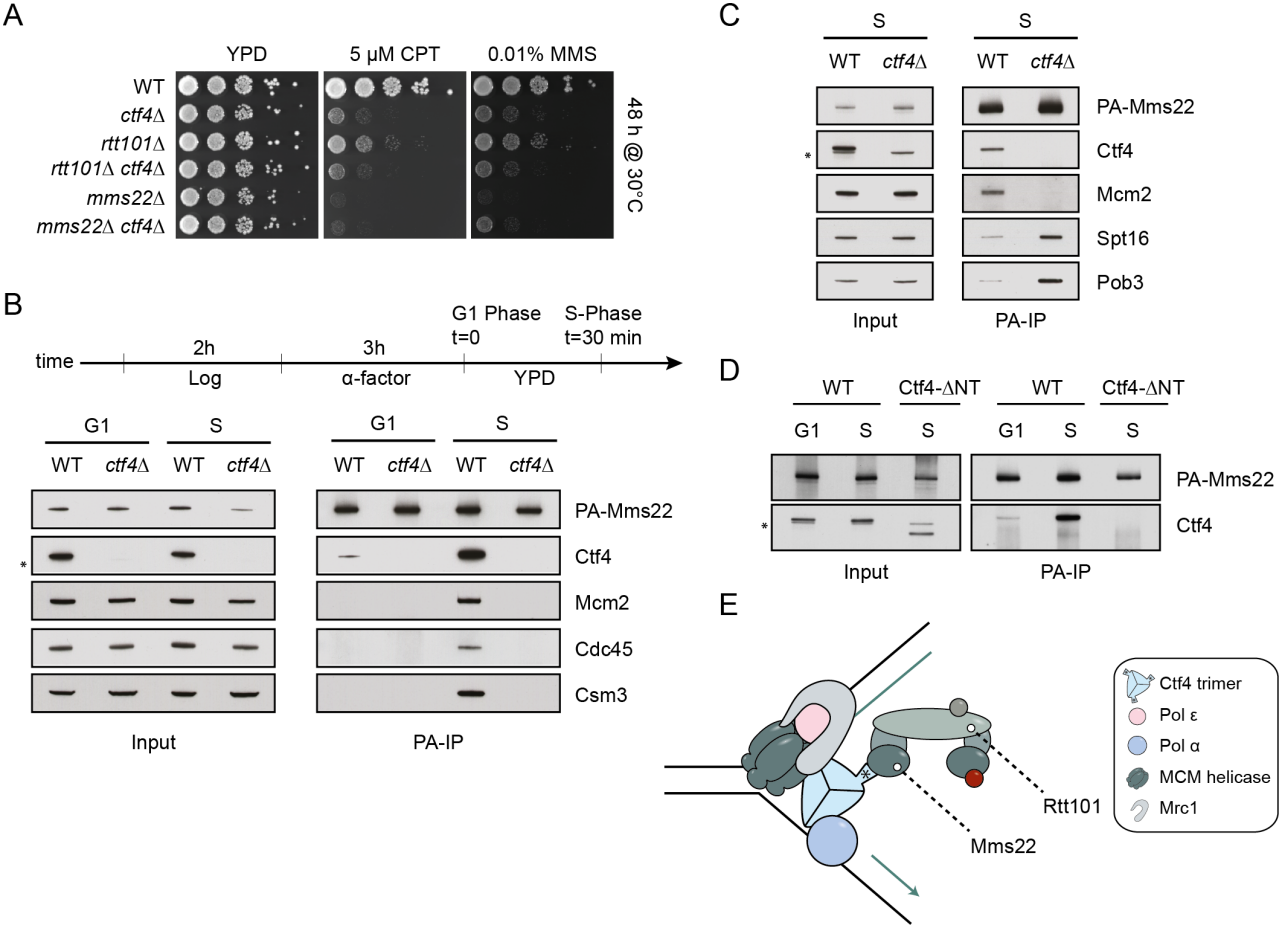
The WD40 domain of Ctf4 recruits the Rtt101^Mms22^ E3 ubiquitin ligase to the replisome progression complex. (A) *CTF4* is epistatic with *RTT101* and *MMS22*. Exponentially growing wild-type (WT), *ctf4Δ*, *rtt101Δ*, *mms22Δ*, *rtt101Δ* *ctf4Δ* and *mms22Δ* *ctf4Δ* cells were spotted in serial dilution on normal growth media (YPD) and media containing 5 μM CPT or 0.01% MMS. The plates were imaged after 48 hours incubation at 30°C. (B) and (C) Ctf4 tethers the Rtt101 E3 ligase to the replisome. Wild-type (WT) and *ctf4Δ* cells were transformed with a plasmid expressing PA-Mms22, arrested in α-factor (G1) or synchronously released into S-phase (S) (diagram). PA-Mms22 was purified from G1 or S-phase extracts and interacting replisome (B) or FACT (C) components were detected using specific antibodies. The asterisk (*) indicates an unspecific band. (D) The WD40 domain of Ctf4 is crucial for Mms22 interaction with the RPC. Wild-type (WT) and *ctf4-ΔNT* cells were synchronized as described in (B). PA-Mms22 was purified from extracts prepared from cells in G1 or S-phase and its interaction with Ctf4 was monitored by immunoblotting with specific antibodies. (E) Model of the Rtt101^Mms22^ E3 ubiquitin ligase interacting with the replisome. Ctf4 together with Mrc1 connects the DNA polymerases to the MCM helicase at replication forks. Mms22 interacts with the amino-terminal domain of Ctf4 (*), which is required to recruit the Rtt101^Mms22^ E3 ubiquitin ligase to active forks.

We next examined how the Rtt101^Mms22^ E3 ligase is recruited to active replisomes. Ctf4 was an intriguing candidate as it was previously found to interact with Mms22 [3,18,36]. Indeed, genetic analysis revealed that the growth defect of *ctf4Δ* cells on genotoxic drugs was epistatic with *RTT101* and even slightly suppressed the sensitivity of *mms22Δ* cells (Fig 3A), consistent with previous findings [36]. To test whether Ctf4 tethers Mms22 to the replisome, we compared the presence of replisome components in Mms22 purifications prepared from wild-type and *ctf4Δ* cells (Fig 3B). Notably, Mms22 failed to interact with the replication factors Mcm2, Cdc45 and Csm3 during S-phase in *ctf4Δ* cells (Fig 3B), while binding to Spt16 and Pob3 FACT complex components was not perturbed (Fig 3C). Mms22 interacts with Ctf4 both by two-hybrid [3,36] and co-immunoprecipitation analysis (Fig 2A and 2C), and requires the amino-terminal WD40 domain of Ctf4 (Figs 3D and S4; [3]). Together, these results suggest that the Rtt101^Mms22^ E3 ligase is tethered to active replisomes via the Ctf4 scaffold (Fig 3E).

### The replicative functions of Mrc1 but not Tof1 or Csm3 are required to suppress toxicity in *rtt101Δ* and *mms22Δ* cells

**Fig 4:**
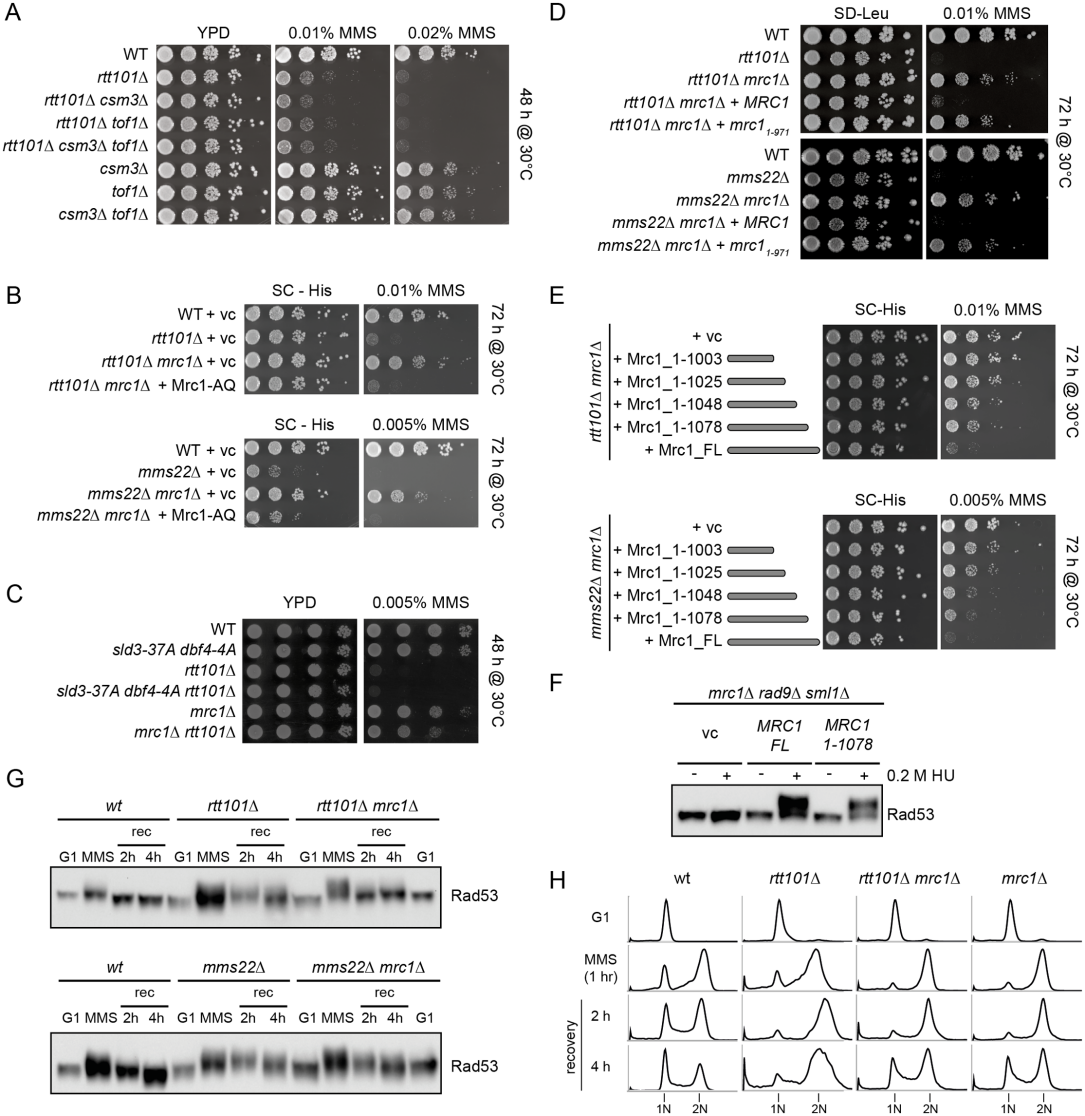
The replicative function of Mrc1 compensates for replication defects in cells lacking components of the Rtt101^Mms22^ E3 ubiquitin ligase. (A) Suppression of the growth phenotype of cells lacking components of the Rtt101^Mms22^ E3 ligase is specific to Mrc1. Serial dilution of wild-type (WT), *rtt101Δ*, *csm3Δ*, *csm3Δ* *rtt101Δ*, *tof1Δ*, *tof1Δ* *rtt101Δ*, *tof1Δ* *rtt101Δ csm3Δ* and *csm3Δ tof1Δ* cells were analyzed on normal growth media (YPD) with or without 0.01% and 0.02% MMS.(B) The checkpoint-defective Mrc1-AQ allele does not suppress the sensitivity of *mms22Δ* or *rtt101Δ* cells to MMS. Serial dilution of wild-type (WT), *rtt101Δ*, *mrc1Δ*, *mms22Δ*, *rtt101Δ mrc1Δ*, *mms22Δ mrc1Δ* strains were assayed on selective growth media (-His) with or without MMS. The lack of *MRC1* was complemented as indicated with plasmids expressing either vector control (vc) or Mrc1-AQ. Plates were imaged after 72 hours incubation at 30°C. (C) De-repressing late origins is not sufficient to suppress *rtt101Δ* MMS sensitivity. Strains with the indicated genotypes were serial diluted on the indicated media as described above. Plates were imaged after 48 hours incubation at 30°C. (D)-(F) C-terminal truncations of Mrc1 can provide a genetic rescue of *rtt101Δ* and *mms22Δ* cells. (D and E) Cells expressing either a vector control (vc), wild-type (FL = full length) or the indicated Mrc1 truncation mutants were spotted on selective growth media (-His) with or without MMS. The black bars represent the c-terminally truncated Mrc1 proteins. Plates were imaged after 72 hours incubation at 30°C. (F) *mrc1Δ rad9Δ sml1Δ* cells were transformed with an empty control vector (vc), or plasmids encoding either wild-type (*MRC1*_*FL*_) or the *MRC1*_*1-1078*_ truncation allele and synchronously released into media with (+) or without (-) 0.2M hydroxyurea (HU). Rad53 phosphorylation was analyzed by immunoblotting of protein extracts with anti-Rad53 antibody. (G) and (H) The checkpoint defect of *rtt101Δ* and *mms22Δ* cells is alleviated by loss of *MRC1*. Cells were synchronized in G1 phase using α-factor and released into media containing 0.01% MMS for 60 min. Stalled forks were then allowed to restart in normal growth media (recovery) and the checkpoint status was monitored by Rad53 phosphorylation (G) and flow cytometry (H) at the indicated time points (in hours (h); rec = recovery).

To determine if the alleviated growth defect observed in *rtt101Δ* *mrc1Δ* strains is phenocopied by the removal of other components of the fork-protection complex (FPC), we tested whether deletion of either *TOF1*, *CSM3* alone or in combination would also rescue the sensitivity of *rtt101Δ* or *mms22Δ* cells to genotoxic agents. Neither the single nor double deletions of *CSM3* and *TOF1* were able to restore growth of *rtt101Δ* or *mms22Δ* cells on media containing MMS (Figs 4A and S5). These data suggest that Rtt101-Mms22 counteracts a function of Mrc1 at replication forks that is independent of its interactions with Tof1 and Csm3.

Since the replication and checkpoint functions of Mrc1 are genetically discernable, we set out to examine which functions of Mrc1 are causing lethality in the absence of Rtt101^Mms22^. We analyzed the genetic interaction between *rtt101Δ* or *mms22Δ* mutants and the well-characterized checkpoint-deficient *MRC1-AQ* allele, coding for a mutant protein in which all Mec1-dependent phospho-serines (SQ) are mutated to alanine (AQ) [37]. Interestingly, when the checkpoint-defective Mrc1-AQ variant is the only source of Mrc1 in cells lacking the Rt101^Mms22^ E3 ubiquitin ligase, the lethal phenotype is comparable to *rtt101Δ* and *mms22Δ* single mutant cells when exposed to genotoxic stress (Fig 4B), unlike the complete deletion of *MRC1* (Figs 1C, 1E and S5). This result was surprising in light of recent data proposing that alleviation of the checkpoint-mediated late origin repression may rescue the sensitivity of *rtt101Δ* cells to MMS exposure [29]. To corroborate these results, we tested whether the requirement of Rtt101 to promote growth on MMS containing media could be bypassed by genetically de-repressing late origins in the *sld3-37A dbf4-4A* background [38]. In contrast to *rtt101Δ mrc1Δ* double mutants, *rtt101Δ sld3-37A dbf4-4A* cells were unable to rescue the sensitivity of *rtt101Δ* cells to MMS (Fig 4C), supporting the notion that Mrc1-dependent inhibition of late origin firing is not sufficient to explain the essential function of the Rtt101^Mms22^ complex in response to genotoxic stress. Conversely, the MMS-induced lethality of *rtt101Δ* or *mms22Δ* cells was suppressed by expressing an Mrc1 C-terminal truncation mutant (Mrc1_1-971_) as the only copy of Mrc1 (Fig 4D). While this mutant has been reported to be checkpoint defective [24], the C-terminal domain of Mrc1 is known to directly interact with the C-terminal domain of Pol2 [13] and is important for the replication functions of Mrc1 [39]. To identify a *bonafide* separation-of-function Mrc1 allele, we thus constructed smaller C-terminal truncations of Mrc1. Strikingly, deletion of only the last C-terminal 18 amino acids (Mrc1_1-1078_) was sufficient to confer a rescue of MMS sensitivity in both *rtt101Δ* and *mms22Δ* cells (Fig 4E, compare bottom two rows), while otherwise checkpoint defective cells expressing the Mrc1_1-1078_ mutant protein were able to activate the replication checkpoint when challenged with replication stress (Fig 4F). Together, these results strongly suggest that loss of Mrc1’s checkpoint function is not responsible for the genetic suppression of *rtt101Δ* or *mms22Δ* cells and implicates the Mrc1 replicative functions in the observed cell toxicity. Interestingly, the inability of *rtt101Δ* or *mms22Δ* cells to extinguish the DNA damage checkpoint following MMS recovery [28] was alleviated in *rtt101Δ mrc1Δ* or *mms22Δ mrc1Δ* strains (Fig 4G), and as a consequence *rtt101Δ mrc1Δ* double mutants proceeded into the next G1 phase four hours post-recovery whereas the *rtt101Δ* cells remained largely arrested at the G2/M border as expected (Fig 4H). Based on these data we conclude that Rtt101^Mms22^ specifically counteracts a replicative function of Mrc1 at stalled replisomes, thereby promoting replication fork repair/restart, which leads to an eventual checkpoint termination.

### *MRC1* deletion compensates for homologous recombination defects in *mms22Δ* cells

**Fig 5:**
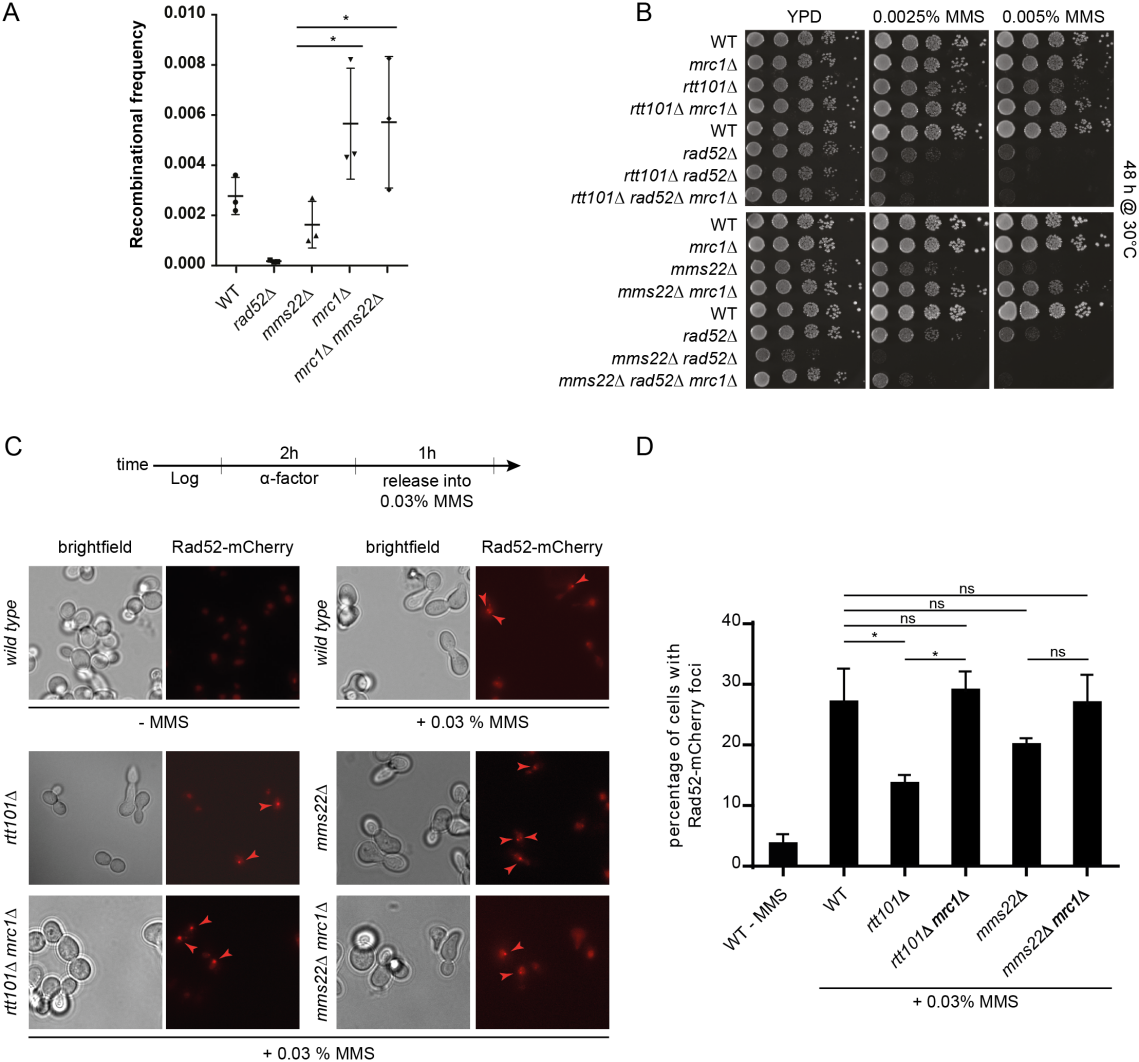
Mrc1 prevents Rad52-mediated HR in *mms22Δ* and *rtt101Δ* cells. (A) Deleting *MRC1* compensates for the HR defect of *mms22Δ* cells. Wild-type (WT) and the indicated mutant strains were transformed with a homologous recombination reporter plasmid (YCpHR), and the recombination frequency (%) was quantified from three independent experiments as schematically outlined in S6 Fig on plates containing 60 μg/ml canavanine. A one-way ANOVA analysis was used for the statistical analysis. * represents a significant difference with 95% confidence. Error bars indicate standard deviation. (B) The growth restoration of *rtt101Δ mrc1Δ* and *mms22Δ mrc1Δ* cells on MMS is *RAD52-*dependent. Serial dilution of wild-type (WT) or *mrc1Δ*, *rtt101Δ*, *rtt101Δ mrc1Δ*, *rtt101Δ mrc1Δ rad52Δ*, *mms22Δ*, *mms22Δ mrc1Δ*, *mms22Δ mrc1Δ rad52Δ* mutants were assayed on normal growth media and media containing 0.0025% or 0.005% MMS and imaged after 48 hours incubation at 30°C. (C) and (D) *rtt101Δ* and *mms22Δ* cells are defective in forming *RAD52* foci. Synchronized cells were released into the presence of MMS as depicted (top diagram) and cells with Rad52-mCherry foci were scored (C). The experiment was performed with two biological replicates, and for each genotype a minimum of 400 cells per replicate were counted (D). * represents a significant difference with 95% confidence intervals following a one-way ANOVA analysis. ns: not significant (below 95% confidence). Error bars indicate standard deviation.

Increasing evidence points towards a key HR function during replication fork restart at stalled replisomes [40]. Since Mrc1 is a known suppressor of HR [11], we hypothesized that its removal may promote restart of damaged replication forks in *rtt101Δ* and *mms22Δ* cells by an HR-dependent mechanism. To assess HR rates we used a previously described reporter system [41] that exploits a plasmid as a recombination substrate for both the single-strand invasion and annealing pathways, resulting in *CAN1* gene deletion (S6 Fig). In agreement with previous studies, HR levels were abolished in *rad52Δ* and reduced in *mms22Δ* strains [42], whereas *mrc1Δ* cells exhibited increased recombination rates compared to wild-type controls ([12] and Fig 5A). Interestingly, *mms22Δ mrc1Δ* double mutants showed a level of HR comparable to *mrc1Δ* cells, suggesting that in the absence of *MMS22*, Mrc1 may block recombination (Fig 5A). In contrast, *ctf4Δ mrc1Δ* double mutants are inviable ([3,43], S7 Fig), implying that Mrc1 and Ctf4 share a Mms22-independent essential function during DNA replication. The increased HR phenotype seems to be specific to Mrc1, as another mutant, *sgs1Δ*, with increased HR levels did not alleviate the genotoxic sensitivity (S8 Fig) or the low HR frequency [34] observed in *mms22Δ* strains. Since the increased HR level correlated positively with the observed genetic suppression in *mms22Δ mrc1Δ* cells, we tested whether HR was required for this suppression by deleting the *RAD52* gene, and thereby rendering cells HR defective. Indeed, the hyper-sensitivity of *rtt101Δ rad52Δ* and *mms1Δ rad52Δ* cells to MMS could no longer be rescued upon further deletion of *MRC1* (Figs 5B, top and S9), consistent with the notion that an intact HR machinery is required for suppression. A similar effect was also observed in cells lacking the substrate adaptor Mms22 (Fig 5B, bottom), but in this case a slight *mrc1Δ* rescue was still observed in the *rad52Δ* background. These data imply that in contrast to *rtt101Δ* and *mms1Δ*, deletion of *MRC1* in *mms22Δ* cells rescues MMS sensitivity, in part, in a *RAD52-*independent manner.

To corroborate these results, we released G1 synchronized cells endogenously expressing mCherry-tagged Rad52 (Rad52-mCherry) into media containing MMS and scored the ability of cells to form Rad52 foci, a proxy for active recombination [44]. As expected, the percentage of cells with Rad52 foci decreased in both *rtt101Δ* and *mms22Δ* cells, but strikingly, this defect was corrected by further deleting *MRC1* (Fig 5C and 5D). Together, these data indicate that HR upregulation induced by the loss of Mrc1 function may play a key role in the restart/repair of defective replication forks in cells lacking Rtt101^Mms22^ E3 ligase activity.

### The stability of Mrc1 is not dependent on the Rtt101^Mms22^ E3 ubiquitin ligase

**Fig 6:**
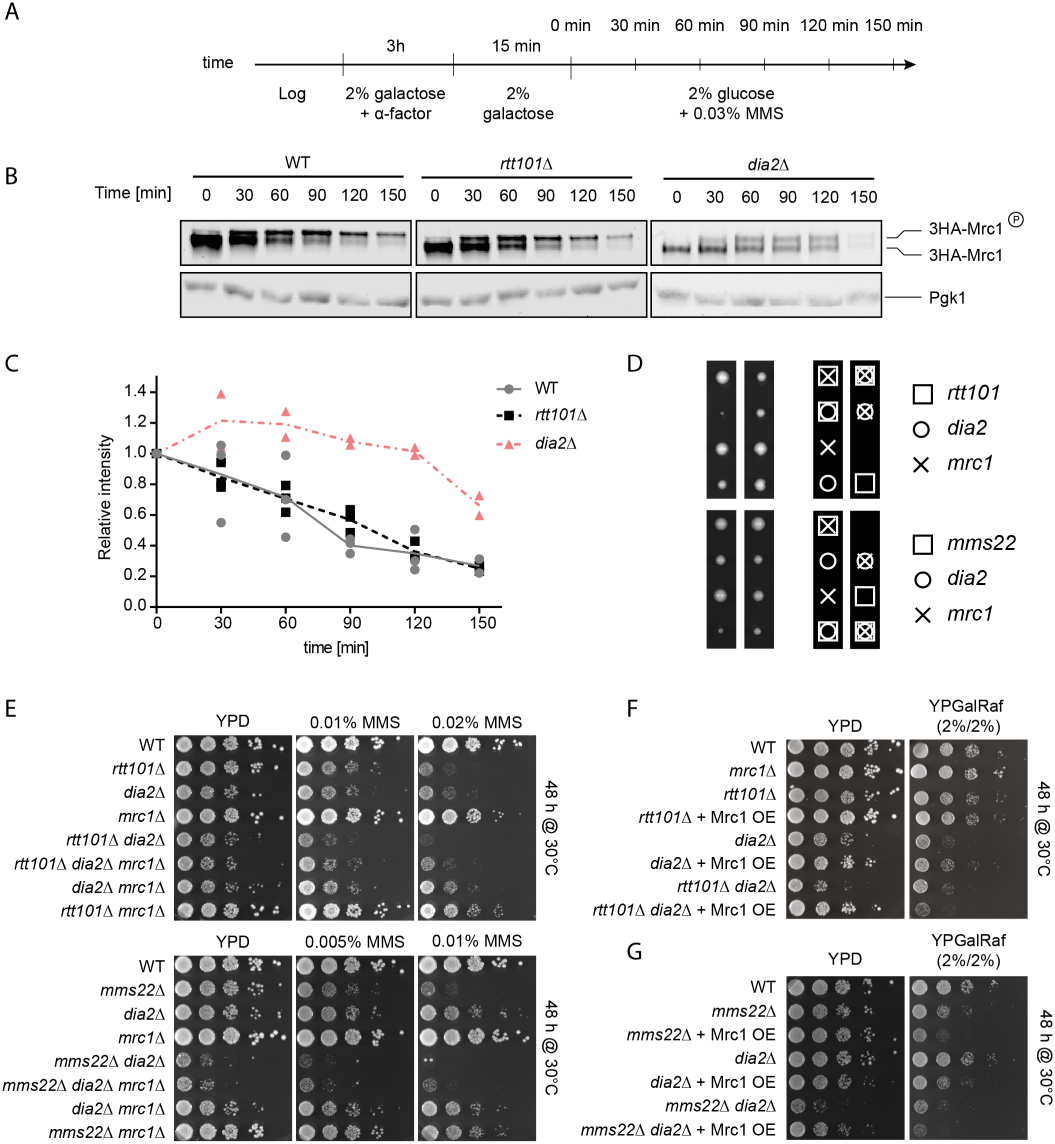
Rtt101^Mms22^ and SCF^Dia2^ counteract Mrc1 activity by distinct mechanisms. (A)–(C) Mrc1 stability is not altered in cells lacking *RTT101*. Wild-type (WT), *rtt101Δ* or *dia2Δ* cells expressing 3HA-tagged Mrc1 from the inducible *GAL1*,*10*-promoter were synchronized in G1 phase using α-factor in 2% galactose and released into S-phase in 2% galactose as outlined in (A). Subsequently, 0.03% MMS and 2% glucose was added to induce fork stalling and repress HA-Mrc1 expression, respectively. Samples were collected at the indicated time points (min) (A), quantified by anti-HA immunoblotting (B) and shown as scatter plots of individual biological replicates with their means (C). Immunoblotting for Pgk1 controls for equal loading. The position of phosphorylated (3HA-Mrc1-P) and unphosphorylated 3HA-Mrc1 is indicated. (D) *RTT101* and *MMS22* are synthetic-sick with *DIA2*. Tetrad analysis from sporulated heterozygote *rtt101Δ dia2Δ mrc1Δ* and *mms22Δ dia2Δ mrc1Δ* diploids. Boxes (□), crosses (X) and circles (O) indicate that the haploid cells lack *RTT101/MMS22*, *MRC1* or *DIA2*, respectively. Note that *rtt101Δ dia2Δ* and *mms22Δ dia2Δ* double mutants indicated by boxes and circles are barely viable. (E) *rtt101Δ dia2Δ* and *mms22Δ dia2Δ* double mutants can be rescued by deletion of *MRC1*. Serial dilution of the indicated strains obtained from the tetrad dissections shown in (D) were assayed on normal growth media (YPD) and media containing 0.02%, 0.01% or 0.005% MMS. The plates were imaged after 48 hours of incubation at 30°C. (F) and (G) Overexpression of Mrc1 is lethal for *rtt101Δ dia2Δ* (F) and *mms22Δ dia2Δ* (G) double mutants. Serial dilution of wild-type (WT) and the indicated mutant strains overexpressing Mrc1 from the galactose-inducible *GAL1*,*10*-promoter were assayed on normal growth media containing 2% glucose (*GAL1*,*10*-promoter off), 2% galactose/2% raffinose (*GAL1*,*10*-promoter on). The plates were imaged after 48 hours of incubation at 30°C.

Available evidence suggests that SCF^Dia2^ targets phosphorylated Mrc1 for proteasomal degradation [22,24]. To test whether Mrc1 is degraded by a Rtt101^Mms22^-dependent mechanism, we monitored Mrc1 stability in synchronized cells with MMS–induced replication stress (Fig 6A). Promoter shut-off by glucose addition to cells expressing galactose-inducible 3HA-Mrc1 (Figs 6A–6C and S10) as well as cyclohexamide (CHX) chase experiments of endogenously tagged 3HA-Mrc1 (S10 Fig) showed that the degradation kinetics of phosphorylated and unphosphorylated Mrc1 in *rtt101Δ* or *mms22Δ* cells exposed to MMS were comparable to wild-type controls. These results were corroborated by quantitative mass spectrometry using selected reaction monitoring (SRM), which demonstrated that Mrc1 levels decreased with similar kinetics in wild-type, *rtt101Δ* and *mms22Δ* cells (S10 Fig). Surprisingly, deletion of *DIA2* had only slight effects on the half-life of Mrc1, and thus the role of SCF^Dia2^ in regulating Mrc1 stability remains to be further clarified [24,45]. Taken together, these data demonstrate that Rtt101^Mms22^ does not trigger degradation of the Mrc1 protein at stalled replication forks.

Tetrad analysis and plating assays revealed that the growth defects caused by loss of Rtt101 and Mms22 together with the loss of Dia2 function were additive, and double mutants were further impaired for growth in the presence and absence of MMS (Fig 6D and 6E). This indicates that the two E3 ligases may function independently and have non-overlapping roles during DNA replication. Interestingly, deletion of *MRC1* restored some of the growth defects of *mms22Δ dia2Δ* and *rtt101Δ dia2Δ* double mutants as shown by tetrad analysis (Fig 6D) as well as spotting assays on MMS containing media (Fig 6E). Conversely, overexpression of Mrc1 resulted in toxicity in *rtt101Δ dia2Δ* and *mms22Δ dia2Δ* double mutants even in the absence of exogenous genotoxic stress (Fig 6F and 6G). Together these results indicate that these two CRLs may genetically interact with Mrc1 function but likely by distinct mechanisms.

### The Rtt101^Mms22^ E3 ligase may modulate the interaction of Mrc1 with the MCM helicase complex upon fork stalling

**Fig 7:**
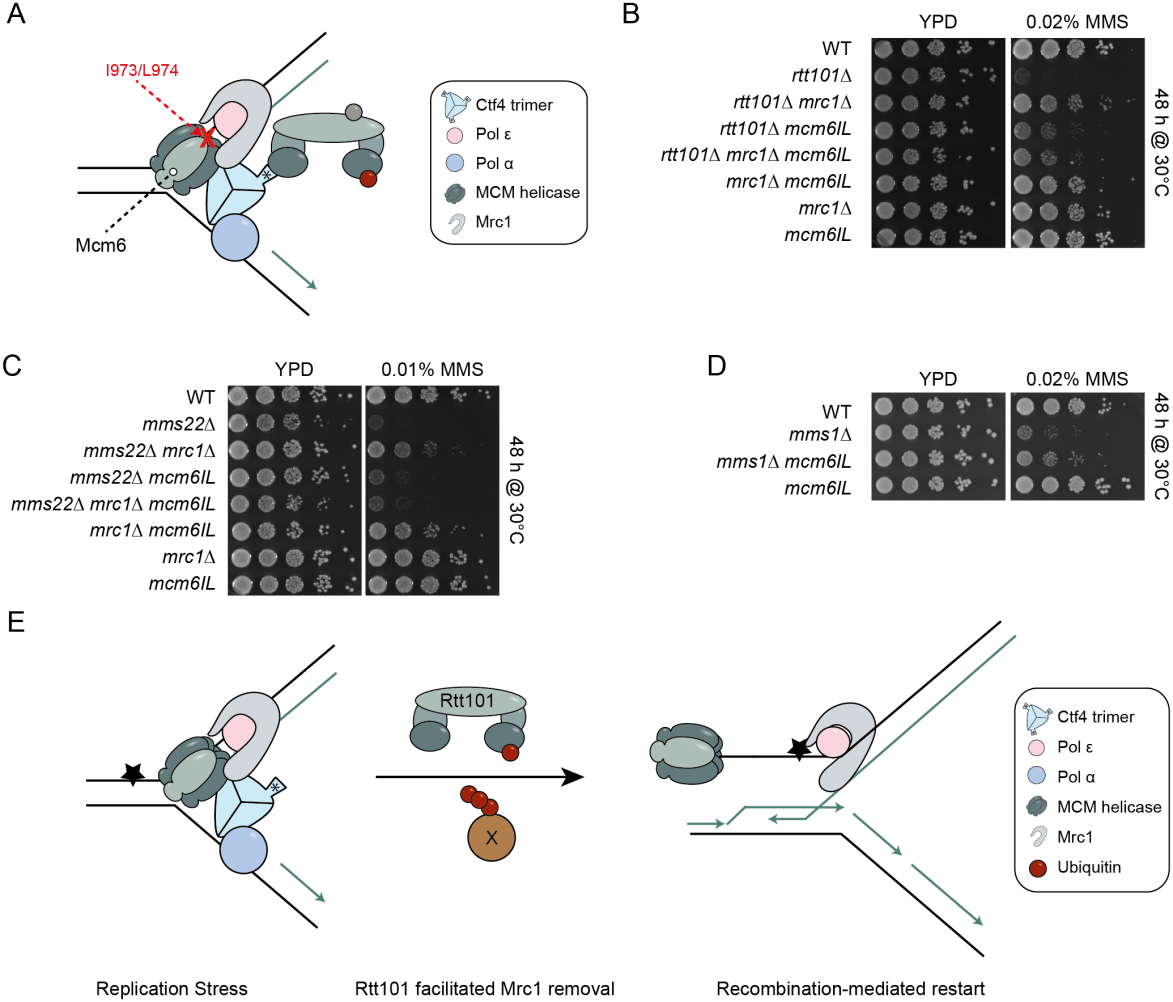
Altered regulation of replicative polymerases and the CMG may allow HR mediated fork restart in cells lacking Rtt101^Mms22^ activity. (A) *mcm6-IL* reduces its affinity to physically interact with Mrc1. Mutation of the indicated amino acids perturbs the interaction between Mcm6 and Mrc1 and is reported to promote uncoupling of the leading strand polymerase from the MCM replicative helicase [14]. The asterisk (*) indicates the WD40 domain of Ctf4. (B)-(D) The *mcm6-IL* allele rescues the genotoxic sensitivity of *rtt101Δ* (B), *mms1Δ* (C) or *mms22Δ* (D) mutants and is epistatic with *MRC1*. Cells with the indicated genotypes were spotted onto either YPD or YPD plates containing 0.02% or 0.01% MMS. Images were taken after 48 hours of incubation at 30°C. (E) Model of Rtt101^Mms22^-facilitated removal of Mrc1 to allow HR-mediated repair/restart of stalled replication forks. The Rtt101^Mms22^ E3 ligase associates with replisomes by binding to Ctf4. When a replication fork encounters an obstacle (star), our data suggest that Rtt101^Mms22^ ubiquitylates a so far unidentified factor (X), which modulates the interaction between Mrc1 and the MCM helicase (Mcm6 is in light grey). This remodeling results in the Rad52-mediated repair/restart of the stressed replisome in order to by-pass the obstacle.

In addition to binding DNA polymerase ε [13], Mrc1 also interacts with the Mcm6 subunit of the MCM2-7 helicase [14]. In order to disrupt the binding of Mrc1 to Mcm6, we crossed the *mcm6-IL* allele [14] into *rtt101Δ*, *mms22Δ* and *mms1Δ* cells deleted for *MRC1*, and compared growth of the resulting single, double and triple mutants treated with DNA damaging agents (Fig 7A). Importantly, we observed that similar to deleting *MRC1* the presence of *mcm6-IL* was able to suppress the sensitivity of *rtt101Δ*, *mms22Δ* (albeit weakly) and *mms1Δ* cells to MMS (Fig 7B–7D), indicating that disrupting the association of Mrc1 with the CMG helicase is sufficient to restore growth of *rtt101Δ*, *mms1Δ*, and in part, *mms22Δ* mutants exposed to genotoxic stress. Importantly, deletion of *MRC1* in *rtt101Δ mcm6-IL or mms22Δ mcm6-IL* cells did not further improve growth on MMS containing media (Fig 7B and 7C), suggesting that these mutations affect the same molecular process. We did not observe a difference in the amount of chromatin bound Mrc1 when comparing wild-type to *rtt101Δ* and *mms22Δ* cells in the absence and presence of MMS, rendering it unlikely that Rtt101^Mms22^ promotes Mrc1 eviction from chromatin at sites of replication stress (S11 Fig). These genetic and biochemical results suggest that the Rtt101^Mms22^ E3 ligase counteracts a function of Mrc1 that is linked to the replicative helicase at stalled replication forks, and possibly modulates the interaction of Mrc1 with the MCM helicase complex upon fork stalling (Fig 7E).

## Discussion

In this study, we have employed genome-wide genetic screening together with a proteomics approach to gain insight into how the Rtt101^Mms22^ E3 ubiquitin ligase regulates stalled replication forks [28]. We demonstrate that Rtt101^Mms22^ is recruited to replisomes during S-phase by interacting with the N-terminal WD40 domain of Ctf4. Moreover, genetic analysis revealed that the introduction of the *mcm6-IL* allele, or deletion of either *MRC1* or the non-essential Pol ε subunit, *DPB4*, can restore viability of *rtt101Δ*, *mms1Δ* and *mms22Δ* cells exposed to various genotoxic stress conditions. Together, our data suggest that the Rtt101^Mms22^ complex acts directly at replisomes to promote repair and restart of stalled replication forks by counteracting a replicative function of Mrc1, and hence promoting HR.

### The Rtt101^Mms22^ complex counteracts Mrc1 to promote HR-dependent repair/restart of stalled replication forks in response to genotoxic stress

The tight association of DNA synthesis with the unwinding activities of the replicative helicase at replisomes is important for faithful DNA replication. Mrc1 represents a plausible candidate to reinforce this association as it physically interacts with both Pol2 as well as the MCM2-7 helicase. Indeed, altering this interaction may lead to exposed stretches of ssDNA, which are vulnerable to nicking and chemical modifications, and if extensive enough, may unleash the replication checkpoint following the association of sufficient replication protein A (RPA) molecules. In accordance, in the presence of MMS we observed more extensive Rad53 phosphorylation in the absence of MRC1 (Fig 4G). Previous studies have shown that *mrc1Δ* cells fail to inhibit late firing origins [6,46], an effect that could be used to bypass the adverse effects of stalled replication forks. Therefore, deletion of *MRC1* might conceivably alleviate the DNA damage sensitivity of *rtt101Δ* and *mms22Δ* cells by allowing the firing of additional origins and hence promoting the completion of replication [29]. In light of our results, however, this possibility seems unlikely given that the loss of Mrc1’s checkpoint functions fails to rescue the defects associated with *rtt101Δ* and *mms22Δ* cells. Moreover, the requirement of the Rtt101^Mms22^ complex in genotoxic stress conditions could not be bypassed by genetically de-repressing late origins using the *sld3-37A dbf4-4A* background [38]. Alternatively, our results suggest that Mrc1 deletion, and in turn replisome uncoupling, may promote HR-mediated fork restart at stalled replication forks [11]. In support of this idea, Rtt101, Mms1 and Mms22 have been demonstrated to stimulate HR, specifically upon exposure to genotoxic agents [42], and we found that deletion of *MRC1* rescued the reduced recombination rates in cells deleted for *MMS22*. Indeed, an intact HR machinery is required for *rtt101Δ mrc1Δ* and to a lesser extent *mms22Δ mrc1Δ* double mutants to grow in genotoxic stress conditions. Moreover, recombination foci visualized microscopically by Rad52-mCherry were decreased in both *rtt101Δ* and *mms22Δ* cells exposed to genotoxic drugs, but were restored by additionally deleting *MRC1*. We thus propose that the Rtt101^Mms22^ E3 ubiquitin ligase promotes HR-mediated repair and restart by counteracting a replicative function of Mrc1 at stalled replication forks. Surprisingly, this role of Mrc1 is not shared with the other subunits of the replisome progression complex Tof1 and Csm3, although they are thought to help stabilize Mrc1 at replication forks. However, Mrc1 also interacts with replisomes by a Tof1/Csm3-independent mechanism [47], perhaps through its interactions with Pol2 and Mcm6. It seems that this Mrc1 pool is sufficient to inhibit HR and may need to be counteracted by the Rtt101^Mms22^ E3 ligase upon fork stalling (Fig 7E).

### The Rtt101^Mms22^ complex counteracts the replicative function of Mrc1 at stalled forks

In contrast to the S-phase checkpoint defective Mrc1-AQ mutant (Fig 4B), expression of a C-terminal truncation mutant of Mrc1 (Mrc1_1-971_) was able to suppress phenotypes linked to both SCF^Dia2^ and Rtt101^Mms22^ E3 ligases ([24], Fig 4D). Importantly, we identified a Mrc1 separation-of-function mutant (Mrc1_1-1078_), which is checkpoint-proficient but likely unable to perform the replicative function of Mrc1 that leads to toxicity when the Rtt101^Mms22^ E3 ligase is impaired. While the exact mechanism underlying this specific defect remains to be elucidated, we propose that the C-terminus of Mrc1 may directly regulate replisome function by binding to either Pol2 or Mcm6. This model might also help explain the higher rate of HR (Fig 5A), as *mrc1Δ* strains leave more unreplicated single stranded DNA stretches at stalled replication forks [11] that require post-replicative HR.

Several mechanisms allow cells to restore stalled replication forks, underlining the importance of this process (reviewed in [19]). Based on our genetic and proteomic analysis, we propose that Rtt101^Mms22^, presumably via a ubiquitylation event, counteracts a replicative function of Mrc1 in order to promote recombination at stalled DNA replication forks (Fig 7E). Since both the *mcm6-IL* allele as well as the deletion of *MRC1* may affect polymerase and helicase activities (Fig 7A), the Rtt101^Mms22^ complex may somehow regulate the activity of these factors at stalled forks. Indeed, our genetic analysis revealed that the requirement of the Rtt101^Mms22^ complex to inhibit the replicative-function of Mrc1 upon genotoxic stress is epistatic to loss of its binding to the MCM helicase, suggesting that the Rtt101^Mms22^ E3 ligase may directly or indirectly modulate the interaction of Mrc1 with the MCM complex. Although we did not observe a difference in the total amount of Mrc1 associated with chromatin following exposure to MMS (S11 Fig), it remains possible that regulation specifically occurs at a small subset of stalled replication forks. Interestingly, a previous study has reported decreased association of Pol ε and Mrc1 with replication forks in *mms1Δ* mutants [48], which may represent a compensatory response. Thus, while we cannot rigorously exclude that Rtt101^Mms22^ regulates Mrc1 via replisome association, we favor a model by which Rtt101^Mms22^-dependent ubiquitination of Mrc1 or an unknown substrate leads to uncoupling of the MCM helicase at the stalled replicon, thereby promoting HR-dependent repair and restart of stalled replication forks (Fig 7E). However, we do not fully understand how Rtt101 or Mms22 interact with either the error prone TLS branch of bypass synthesis or with other means of replication fork restart (e.g. BIR and replication fork regression) [19]. Since Rad52 is synthetically-sick with Mms22 but not Rtt101 in unchallenged conditions (Fig 5B), it is conceivable that Mms22 may regulate either TLS or replication fork regression, as part of its Rtt101-independent functions. Future studies will be required to test these possibilities.

## Material and Methods

### Yeast strains, plasmids and growth conditions

Plasmids and yeast strains are listed in S3 and S4 Tables, respectively. Standard methods were used for yeast strain construction and molecular biology. Yeast cells were grown in rich medium (YPD; 1% yeast extract, 2% peptone, 2% glucose) or synthetic medium (SD; 0.17% yeast nitrogen base, 0.5% ammonium sulphate, 2% glucose, amino acids as required). Homologous recombination frequencies were measured as described [41]. For spotting assays, the indicated strains were grown overnight at 30°C, and the cultures were diluted to OD_600_ 0.5. Ten-fold serial dilutions were spotted using a pinning head (2 μl). The plates were incubated at 30°C and imaged using the ChemiDoc Touch Imaging System (Bio-Rad) after 2 and 3 days. For cell cycle synchronization, logarithmically growing cells were treated with 1:1000 α-factor solution (5 mg/ml + 0.1% BSA) at 24°C for 3 hours. G1 arrest was monitored by flow cytometry and microscopy (appearance of pear-shaped “shmoo” morphology of at least 95% of cells). Cells were then washed three times with YPD at room temperature and S-phase samples were collected 30 minutes after the release into fresh YPD medium.

### SGA screening and hit validation

Synthetic Genetic Array (SGA) methodology was used as described [30], with the following modifications: the non-essential heterozygous diploid *S*. *cerevisiae* knockout collection (kindly provided by M. Knop) was sporulated and crossed to a *rtt101*::*NAT can1*::*STE2pr-SpHis5* strain (Y7092, C. Boone). Diploids were selected by repinning on YPD plates containing 100 μg/ml nourseothricin and 250 μg/ml of the kanamycin analogue G418. After sporulation, haploid double mutants were selected by repinning on MATa selection plates (SD-his/arg/lys + canavanine + thiolysine) followed by a repinning on MATa selection plates containing 100 μg/ml nourseothricin and 250 μg/ml G418. Colonies were then re-pinned onto SD complete, SD + 0.01% MMS and SD + 5 μM CPT, and repinned twice onto the same media after 24 h incubation at 30°C. Pictures of the last repinning were taken after 24 h incubation at 30°C. The occurrence of suppressors, i.e. double mutants that showed increased resistance to either MMS or CPT, were scored manually, and validated by tetrad analysis from independent starter strains followed by duplicate spotting assays onto drug containing media.

### DNA content quantifications using flow cytometry

Culture volumes of exponentially growing cells corresponding to 0.68 OD units were collected by centrifugation (3000 rpm for 5 min at RT), resuspended in 1 ml cold 70% ethanol and stored at 4°C. Cells were washed once in 1 ml H_2_O (3000 rpm for 5 min at RT), resuspended in 0.5 ml 50 mM Tris-HCl (pH 8.0) and incubated with 10 μl RNase A (10 mg/ml) for 3 h at 37°C. After centrifugation (3000 rpm for 5 min at RT) cells were resuspended in 0.5 ml 50 mM Tris-HCl (pH 7.5) containing 1 mg/ml Proteinase K and incubated for 45 min at 50°C. Cells were spun down (3000 rpm for 5 min at RT) and resuspended in 0.5 ml 50 mM Tris-HCl (pH 7.5). 100 μl of cells were sonicated five times 15 sec at low intensity using the Bioruptor Twin XD10. 50 μl of cells were mixed with 1 ml 1 x SYTOX Green (Life Technologies) in 50 mM Tris-HCl (pH 7.5) to stain DNA. Cells were kept dark and analyzed immediately for DNA content using a BD FACSCanto II flow cytometer using the following filters and settings: FSC and SSC were detected with a 488 nm laser with detector settings of 318 V and 360 V, respectively. SYTOX Green was detected with a 502 nm longpass filter and 530/30 nm bandpass filter at 466 V. 20000 events per sample were analyzed in each run. Data collection and analysis was performed using BD FACSDiva software and FlowJo v10.0.6 (Miltenyi Biotec) software.

### Yeast protein extraction, SDS-PAGE, western blotting and antibodies

2 OD_600_ units of exponentially growing cells were pelleted at 13’000 rpm for 2 min, and if necessary stored at -20°C. Cell pellets were resuspended in 150 μl of Solution 1 (0.97 M 2-mercaptoethanol, 1.8 M NaOH) and incubated on ice for 10 min. 150 μl of Solution 2 (50% TCA) were added, cells were incubated 10 min on ice and centrifuged at 13’000 rpm for 2 min at 4°C. Pellets were resuspended in 1 ml acetone, centrifuged at 13’000 rpm for 2 min at 4°C and the pellets resuspended in 100 μl urea buffer (120 mM Tris-HCl pH 6.8, 5% glycerol, 8 M urea, 143 mM 2-mercaptoethanol, 8% SDS, bromophenol blue indicator). Protein extracts were incubated 5 min at 55°C, centrifuged at 8’000 rpm for 30 sec, separated by SDS-PAGE and transferred onto nitrocellulose. Membranes were blocked with 5% milk and 1% BSA and incubated with appropriate antibodies: Rabbit peroxidase anti-peroxidase (1:10000), mouse monoclonal antibody against c-Myc (1:3000), HA (1:3000), Mcm2 (1:2000), Pgk1 (1:200000), Rad53 (1:16, EL7.E1, gift from M. Foiani), mouse polyclonal antibody against Orc6 (1:500, gift from H. Ulrich). Replisome antibodies are from sheep polyclonal antiserum: Ctf4 (1:2000), Cdc45 (1:1000), Mcm6 (1:1000), Sld5 (1:1000), Psf2 (1:250), Psf3 (1:3000), Csm3 (1:1000), Spt16 (1:3000), Pob3 (1:3000).

### Acetate-salt based replisome affinity purifications

Cell harvesting was performed at 3000 rpm (Multifuge 3 5-R) for 3 min at RT. Samples were first washed with 20 mM Tris-acetate pH 9.0, then with lysis buffer (75 mM (or 100 mM in Fig 2C and 2D) Tris-acetate pH 9.0, 50 mM KOAc, 10 mM MgOAc, 2 mM EDTA, 2 mM NaF, 2 mM β-glycerophosphate, 1× Roche protease inhibitor cocktail, 1× sigma inhibitors). The cell pellets’ mass was weighted and re-suspended in 3 volumes of lysis buffer. The cell suspension was shock-frozen in liquid nitrogen as “droplets” and stored at -80°C. All cell manipulations and collection were performed at 4°C, if not specified otherwise. Equal weight of “droplets” was grinded with a cryogenic impact mill (Freezer-mill 6870 Large SamplePrep), using 5 min pre-cool followed by 6 cycles of 2 min milling at 12 CP and 2 min cooling down. Cells were thawed for 5 min at RT and 0.25 volume of glycerol mix (75 mM (or 100 mM in Fig 2C and 2D) Tris-acetate pH 9.0, 300 mM KOAc, 50 mM MgOAc, 2 mM EDTA, 0.5% NP40, 1 mM DTT, 2 mM NaF, 2 mM β-glycerophosphate, 1× Roche protease inhibitors, 1× yeast inhibitors) was added to the lysate. DNA was digested by 800 Units/ml DNA nuclease (Benzonase Novagen) at 4°C for 30 min, followed by 30 min centrifugation at 15’000 rpm (25’000g) at 4°C (Sorvall RC26 Plus, SS-34 rotor) and 60 min ultracentrifugation at 25’000 rpm (100’000g) at 4°C (Beckman Coulter Optima LE80K, SW-41 rotor) to remove insoluble material. From the resulting extract, 50 μl was used as whole cell extract (WCE) and the remaining was used for affinity-precipitation. 50 μl of WCE was dissolved in 100 μl 1.5×SDS-loading buffer (1× buffer: 50 mM Tris-Cl pH 6.8, 100 mM DTT, 2% SDS, 0.1% bromophenol blue, 10% glycerol) and boiled at 95°C for 5 min. 4 μl of sample was used to load on a Bis-Tris acrylamide gel. Washed IgG coupled dynabeads (M-270 Epoxy; 14302D, Life Technologies) were added to extracts for immuno-precipitation. Samples were incubated for 2 hours on a rotating platform at 4°C. Beads were washed 4 times at RT with 1 ml wash buffer (100 mM Tris-acetate pH 9.0, 100 mM potassium acetate, 10 mM magnesium acetate, 2 mM EDTA) and protein was eluted with 50 μl of 1× SDS-loading buffer.

### Proteomics

#### In-gel trypsinization and protein identification

Acrylamide gel bands were minced into pieces, reduced in Farmer’s solution (30 mM Potassium Ferricyanide (III), 30 mM sodiumthiosulfate) for 2 min and washed until recovering transparency. Gel slices were then incubated with 100 mM ammonium bicarbonate (ABC) for 20 min, washed with 50 mM ABC/50% acetonitrile (ACN), and rinsed with 100% ACN. Gel slices were subsequently dehydrated with 100% ACN for 20 min under gentle agitation and then rehydrated with 100 mM ABC containing 10 mM dithiothreitol (DTT) for 30 min at 56°C. Finally, gel slices were treated with 100 mM ABC containing 50 mM iodoacetamide (IAA) for 30 min at RT. After second dehydration with 100% ACN, the dried gel pieces were rehydrated with 0.02 μg/μl trypsin in 50 mM ABC and incubated 60 min on ice and then over night at 37°C. For the extraction, peptides were sonicated three times in 50% ACN containing 5% formic acid for 10 min at 4°C and dried in a vacuum centrifuge.

Peptide samples were subjected to a label-free, shotgun proteomic analysis on a 5600 TripleTOF (ABSciex, Concord, Canada) with a nano-electrospray ion source. For the chromatographic separation of the peptides, the instrument was coupled with an Eksigent Nano LC system (ABSciex, Foster City, CA, USA) and equipped with a 15-cm fused silica column (BGB Analytik, Böckten, Switzerland), packed in-house with Magic C18 AQ, 5 μm beads (Michrom Bioresources, Leonberg, Germany). Samples of ~3 μg each were loaded from an autosampler at 4°C and separated with a linear gradient of acetonitrile/water containing 0.1% formic acid from 5 to 35% acetonitrile in 120 min, with a flow rate of 300 nl/min. The mass spectrometer was operated in data-dependent acquisition mode, with the accumulation time for TOF scans set to 0.299995 s, and the mass range to 400–1250 Da. Peptides with 2–5 charges and signals exceeding 150 cps were selected for fragmentation. The product ion scan was performed with an accumulation time of 0.149998 s, and a mass range of 170–1500 Da in a high sensitivity mode. The total cycle time was 3.35 s.

The collected spectra were searched against the *Saccharomyces cerevisiae* SGD protein database with Sorcerer-SEQUEST (Thermo Electron, San Jose, CA, USA). Trypsin was set as the digesting protease with the tolerance of two missed cleavages, one non-tryptic terminus and not allowing for cleavages of KP and RP peptide bonds. Protein identifications were statistically analyzed with ProteinProphet (v3.0) and filtered to a cutoff of 0.9 ProteinProphet probability, which in this case corresponds to a FDR<1%, calculated based on a target-decoy approach. The data was used to generate a protein identification list.

#### In-solution trypsinization and SRM analysis

Protein samples were dissolved to a final concentration of 1–3 mg/ml in corresponding lysis buffer (0.1 M NH_4_HCO_3_, 7 M Guanidinium) and sonicated for 5 min. Disulfide bonds were reduced in 12 mM DDT for 30 min at 32°C under agitation. Iodoacetamide (IAA) was then added to a final concentration of 40 mM and samples were incubated under agitation in the dark for 45 min at 25°C, to alkylate free cysteine residues. Samples were diluted to a final concentration of 0.5 M Guanidinium with freshly prepared 0.1 M NH_4_HCO_3_. Sequencing Grade Modified Trypsin (Promega) was added to an enzyme/substrate ratio of 1/50 (w/w) and the reaction mixture incubated at 37°C overnight. The digestion was stopped with formic acid to a final pH <3. The peptide mixtures were applied onto Sep-Pak tC18 cartridges (Waters), desalted according to the manufacturer instructions and eluted with 50% acetonitrile (ACN). Peptide samples were evaporated on a vacuum centrifuge to dryness and re-solubilized in 0.1% formic acid for LC-MS analysis. For the quality control of transitions and retention time estimation, samples were measured in SRM mode on a triple-quadrupole/ion-trap mass spectrometer (5500 QTrap, ABSciex, Concord, Canada) with a nano-electrospray ion source. The instrument was coupled to an Eksigent 1D-plus Nano liquid chromatography system with a 20 cm fused-silica column with 75 μm diameter packed with Magic C18 AQ 5 μm beads for on-line chromatographic separation of peptides. Approximately 1 μg of sample was separated with a linear gradient from 5% to 35% ACN in 30 min. The SRM measurements were conducted with Q1 and Q3 operated at unit resolution (0.7 m/z half-maximum peak width) with a dwell time of 20 ms and cycle time of < 3.0 s.

Data were analyzed with Skyline to define the best transitions, and 3–4 transitions per peptide were retained based on retention time and relative fragment-ion intensity for the final SRM assay. Five peptides were measured for Mrc1 (e.g APEQNHNNGK, CITLDLDSDSDEYGDDDMDSIK, IAINLGHYGDNIGEDTDK, SSAFFESMVEDIIEYK, SVELNLTDETR). For time-scheduled experiments a LC-SRM method was used for peptide measurements with 3 min window, 30 min gradient using the same MS parameters as for SRM assay validation. After data acquisition, the sum of the area corresponding to each selected transition was used to evaluate relative peptide abundances.

### Fluorescence microscopy

At the indicated time points, cells expressing Rad52-mCherry (0.08 OD_600_ units) were pelleted at 3’000 rpm for 3 min, resuspended in 300 μl SD-Trp containing 0.03% MMS, and then transferred into one chamber of a Nunc Lab-Tek coverglass (Thermo Fisher Scientific) coated with 2 mg/ml Concanavalin A (Sigma Aldrich). Images were obtained on a Leica AF7000 widefield microscope using a 63x/1.4 oil objective. Brightfield and fluorescent images were taken along the z-axis, and Rad52-mCherry foci counted in all focal planes for at least 400–600 cells per strain (n = 2 biological replicates).

### Chromatin association assay

G1-arrested cell cultures were split and released into YPD media containing DMSO solvent or 0.03% MMS. S-phase cells were collected after 30 min (untreated) or 1 hour (MMS-treated) at 30°C, stopped with 0.1% sodium azide and harvested at RT by centrifugation for 5 min at 4000 rpm. The pellet was resuspended in 1.5 ml pre-spheroplasting buffer (100 mM PIPES, pH 9.4, 10 mM DTT, 0.1% sodium azide), pelleted again after 10 min at room temperature, and resuspended in 1 ml spheroplasting buffer (50 mM potassium phosphate buffer, pH 7.5, 0.6 M sorbitol, 10 mM DTT, 0.2 mg/ml zymolyase (>200 units/mg)). After 1 h incubation at 30°C, spheroplasts were spun down at 4°C for 1 min at 2500 rpm, washed with 1 ml wash buffer (100 mM KCl, 50 mM HEPES-KOH, pH 7.5, 2.5 mM MgCl_2_, 0.4 M sorbitol) and resuspended in 100 μl extraction buffer (100 mM KCl, 50 mM HEPES-KOH, pH 7.5, 2.5 mM MgCl_2_, 1× Roche protease inhibitor cocktail). The suspension was split into three aliquots of 50 μl each (whole cell extract, soluble fraction, chromatin bound fraction), cells lysed by adding 0.25% Triton X-100 and 5 min incubation on ice, and the cell extract treated with 1 μl of a 1:50 dilution of benzonase (NEB). After 15 min incubation on ice, NuPAGE LDS sample buffer was added, the soluble fraction centrifuged at 4°C for 10 min at 14000 rpm, and the supernatant transferred to a new reaction tube. The chromatin bound fraction was underlayed with 30% sucrose solution and centrifuged at 4°C for 10 min at 14000 rpm. The supernatant was discarded and the pellet resuspended in 50 μl extraction buffer with 0.25% Triton X-100. This was repeated once, the resuspended final pellet treated at 4°C with 1 μl of a 1:50 dilution of benzonase, and the reaction stopped after 15 min by the addition NuPAGE LDS sample buffer. All samples were incubated for 10 min at 70°C, cleared by centrifugation for 10 min at 13000 rpm, and the samples analyzed by immunoblotting using 4–15% pre-cast polyacrylamide gels (Bio-Rad).

## Supporting Information

S1 Fig Exemplary plates from the synthetic genetic array (SGA) screening. Genotoxic conditions included MMS (0.01%) and CPT (5 μM). Plates were imaged after 72 hours at 30°C. The boxes highlight double mutants (pinned in quadruplicate) where genetic suppression was detected.
(TIF)

S2 Fig Genotoxic sensitivity of the neddylation-deficient *RTT101-K791R* allele is alleviated in cells lacking *MRC1*. Serial dilution of *rtt101Δ* or *rtt101Δ mrc1Δ* cells transformed with plasmids containing either *RTT101* or *RTT101-K791R* were analyzed on selective growth media (SD-His) with or without 10 μM CPT or 0.01% MMS. The plates were imaged after 48 hours of incubation at 30°C (A). Expression of CBP-9Myc-tagged Rtt101 and Rtt101-K791R was monitored by immunoblotting with anti-myc antibodies (B). The slower migrating band marks neddylated, active Rtt101 (CBP-9Myc-Rtt101^Nedd8^).
(TIF)

S3 Fig Deletion of *MRC1* does not suppress the genotoxic sensitivity of cells lacking *RAD52* or *UBC13*. Serial dilution of wild-type (WT) or *rtt101Δ*, *mrc1Δ*, *mrc1Δ rtt101Δ*, *rad52Δ*, *mrc1Δ rad52Δ*, *ubc13Δ* and *mrc1Δ ubc13Δ* mutant strains were assayed on normal growth media (YPD) or media containing MMS (0.005%, 0.01%, 0.02%). The plates were imaged after 48 hours of incubation at 30°C.
(TIF)

S4 Fig Domain structure of Ctf4. Schematic drawing of Ctf4, with its WD40, beta propeller and alfa-helical domains. The numbers indicate the amino-acids starting with the amino-terminal methionine. The amino-terminally truncated Ctf4-ΔNT mutant (encompassing amino acids 461–927) unable to interact with Mms22 is indicated below.
(TIF)

S5 Fig Suppression of the growth phenotype of cells lacking components of the Rtt101^Mms22^ E3 ligase is specific to Mrc1. Serial dilution of wild-type (WT) or *mrc1Δ*, *tof1Δ*, *csm3Δ*, *mms22Δ*, *mms22Δ mrc1Δ*, *mms22Δ tof1Δ and mms22Δ csm3Δ* cells were analyzed on normal growth media (YPD) with or without 0.01% MMS. The plates were imaged after 48 hours of incubation at 30°C.
(TIF)

S6 Fig Homologous recombination reporter assay. (A) Schematic representation of the YCpHR plasmid reporter [41] used in Fig 5A. (B) Cells transformed with the YCpHR reporter were grown for 5 hours in normal growth conditions (SD-Leu) and plated on either SD-Leu or SD–Leu + canavanine (CAN) media to assess the recombination frequency. CAN resistant colonies were quantified after 72 hours using a SegmentColonies Matlab script.
(TIF)

S7 Fig *MRC1* cells are synthetic-lethal with *CTF4*. Tetrad analysis from sporulated heterozygote *ctf4Δ mrc1Δ* diploids. Crosses (X) and circles (O) indicate haploid cells lacking *MRC1* or *CTF4* respectively.
(TIF)

S8 Fig Deletion of *MRC1* does not suppress the genotoxic sensitivity observed in cells lacking *SGS1*. Serial dilution of wild-type (WT) or *mms22Δ*, *mrc1Δ*, *mms22Δ mrc1Δ*, *sgs1Δ*, *mms22Δ sgs1Δ* mutants were analyzed on normal growth media (YPD) with or without 0.005% MMS or 5 μM CPT. The plates were imaged after 48 hours of incubation at 30°C.
(TIF)

S9 Fig The growth restoration of *mms1Δ mrc1Δ* cells on MMS is *RAD52* dependent. Serial dilution of wild-type (WT) or *mrc1Δ*, *mms1Δ*, *mms1Δ mrc1Δ* and *mms1Δ mrc1Δ rad52Δ* mutants were assayed on normal growth media and media containing 0.0025% or 0.005% MMS. The plates were imaged after 48 hours of incubation at 30°C.
(TIF)

S10 Fig Mrc1 stability is not altered in cells lacking *RTT101* or *MMS22*. *mms22Δ* cells expressing 3HA-tagged Mrc1 from the inducible *GAL1*,*10*-promoter were synchronized in G1 phase using α-factor in 2% galactose and released into S-phase in 2% galactose as outlined in Fig 6A. Subsequently, 0.03% MMS and 2% glucose was added to induce fork stalling and repress HA-Mrc1 expression, respectively. Samples were collected at the indicated time points (min) and HA-Mrc1 levels monitored by anti-HA immunoblotting (A). Immunoblotting for Pgk1 controls for equal loading. The position of phosphorylated (3HA-Mrc1-P) and unphosphorylated 3HA-Mrc1 is indicated. In an independent approach, wild-type (WT), *mms22Δ* and *rtt101Δ* cells expressing 3HA-tagged Mrc1 were synchronized in G1 phase using α-factor in YPD and released into S-phase in YPD + 0.03% MMS as outlined in (B). After 40 min, cells were released in normal growth media containing 200 μg/ml cycloheximide (CHX) and 3HA-Mrc1 was detected at the indicated times by immunoblotting with HA-antibodies (C). The position of phosphorylated (3HA-Mrc1-P) and non-modified (3HA-Mrc1) is marked. Mrc1 protein levels were quantified and normalized from two independent experiments. In addition, endogenous, untagged Mrc1 levels in wild-type (WT), *rtt101Δ* and *mms22Δ* were independently quantified by selective-reaction-monitoring (SRM) by measuring transitions corresponding to 5 independent Mrc1 peptides (**D**). Relative intensities are indicated with standard deviations from five independent peptide measurements. Note that Mrc1 is degraded after release from genotoxic stress by a Rtt101^Mms22^-independent mechanism.
(TIF)

S11 Fig Chromatin association of Mrc1 is not altered in *rtt101Δ* or *mms22Δ* cells both in the absence and presence of damage. Mrc1-myc expressing strains were synchronized in G1 phase using α-factor and released into medium with (+) or without (-) 0.03% MMS. S-phase samples were collected and the chromatin-bound proteins were separated from the soluble fraction (for detailed experimental procedure see Materials and Methods section). The presence of Mrc1-myc as well as chromatin-associated Orc6 and the soluble Pgk1 controls were detected by immunoblotting in whole cell extract (WCE), the chromatin-associated fraction (pellet = P) and the soluble fraction (supernatant = Sup).
(TIF)

S1 Table List of candidates identified in the SGA screen. (XLSX)

S2 Table List of Mms22 interactors. PA-tagged Mms22 was immunoprecipitated from cells synchronized in S-phase and associated proteins were identified by LC-MS/MS. The percentage (%) coverage of each associated protein is indicated.
(XLSX)

S3 Table List of plasmids used in this study. (XLSX)

S4 Table List of yeast strains used in this study. (XLSX)
