# Supplementary figures and images for: The Replisome-Coupled E3 Ubiquitin Ligase Rtt101Mms22 Counteracts Mrc1 Function to Tolerate Genotoxic Stress
Source: PLoS Genet. 2016 Feb 5;12(2):e1005843. doi: 10.1371/journal.pgen.1005843 (PMC4743919; doi:10.1371/journal.pgen.1005843)

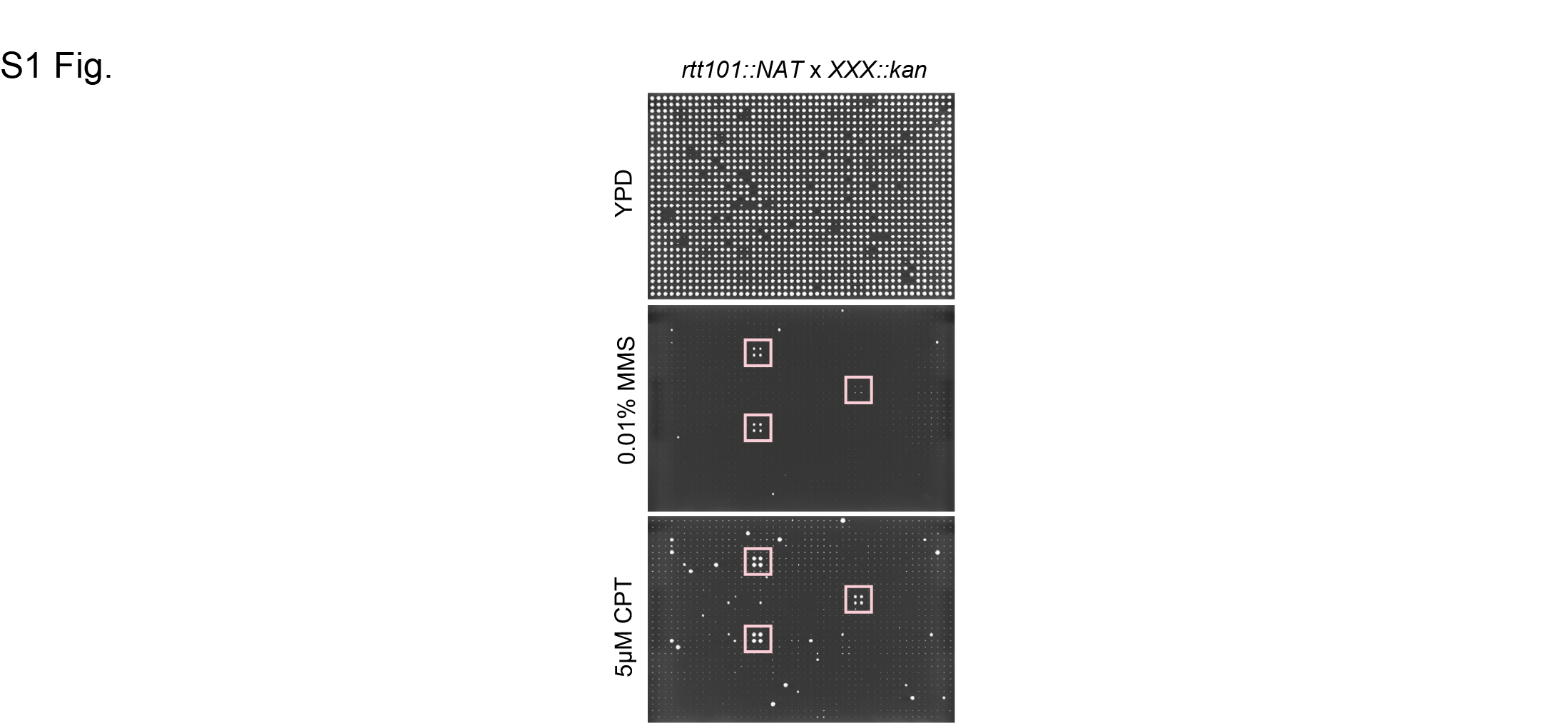

Supplement: S1 Fig — Genotoxic conditions included MMS (0.01%) and CPT (5 μM). Plates were imaged after 72 hours at 30°C. The boxes highlight double mutants (pinned in quadruplicate) where genetic suppression was detected. (TIF) [file pgen.1005843.s001.tif]

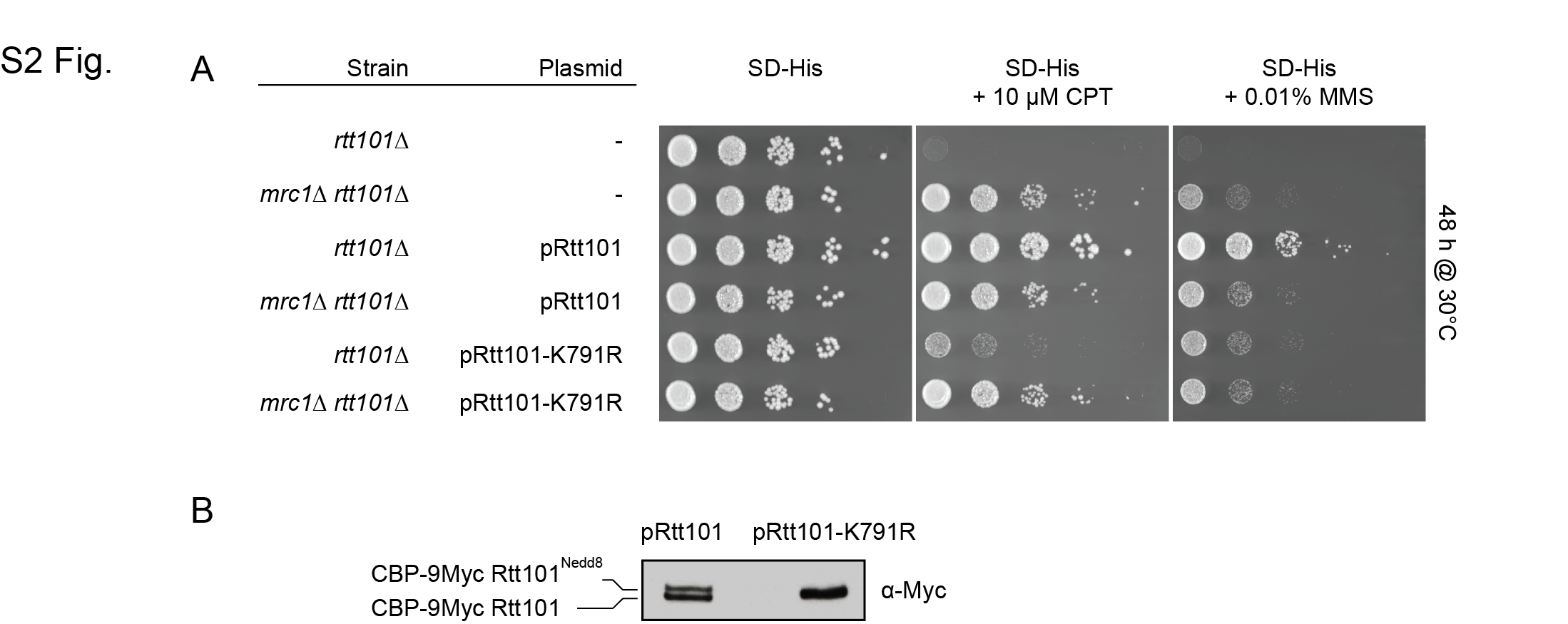

Supplement: S2 Fig — Serial dilution of rtt101Δ or rtt101Δ mrc1Δ cells transformed with plasmids containing either RTT101 or RTT101-K791R were analyzed on selective growth media (SD-His) with or without 10 μM CPT or 0.01% MMS. The plates were imaged after 48 hours of incubation at 30°C (A). Expression of CBP-9Myc-tagged Rtt101 and Rtt101-K791R was monitored by immunoblotting with anti-myc antibodies (B). The slower migrating band marks neddylated, active Rtt101 (CBP-9Myc-Rtt101Nedd8). (TIF) [file pgen.1005843.s002.tif]

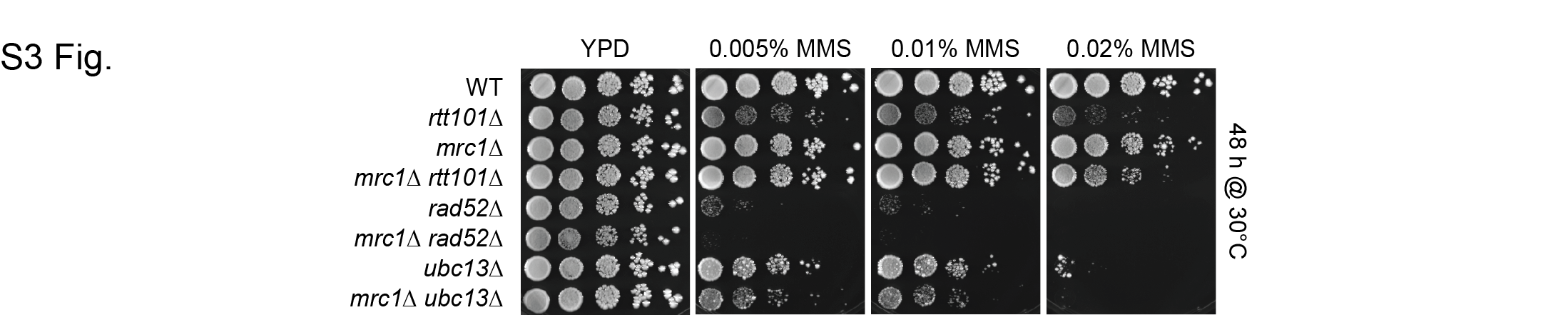

Supplement: S3 Fig — Serial dilution of wild-type (WT) or rtt101Δ, mrc1Δ, mrc1Δ rtt101Δ, rad52Δ, mrc1Δ rad52Δ, ubc13Δ and mrc1Δ ubc13Δ mutant strains were assayed on normal growth media (YPD) or media containing MMS (0.005%, 0.01%, 0.02%). The plates were imaged after 48 hours of incubation at 30°C. (TIF) [file pgen.1005843.s003.tif]

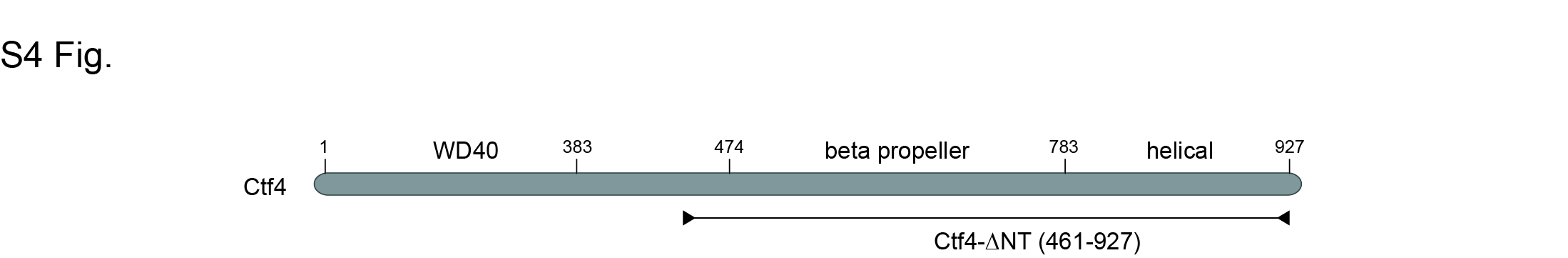

Supplement: S4 Fig — Schematic drawing of Ctf4, with its WD40, beta propeller and alfa-helical domains. The numbers indicate the amino-acids starting with the amino-terminal methionine. The amino-terminally truncated Ctf4-ΔNT mutant (encompassing amino acids 461–927) unable to interact with Mms22 is indicated below. (TIF) [file pgen.1005843.s004.tif]

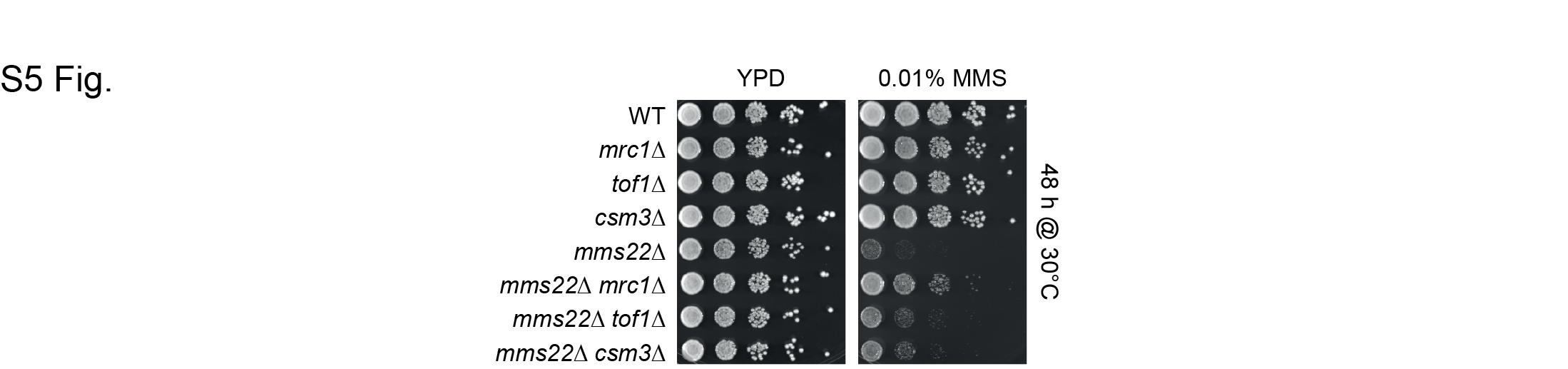

Supplement: S5 Fig — Serial dilution of wild-type (WT) or mrc1Δ, tof1Δ, csm3Δ, mms22Δ, mms22Δ mrc1Δ, mms22Δ tof1Δ and mms22Δ csm3Δ cells were analyzed on normal growth media (YPD) with or without 0.01% MMS. The plates were imaged after 48 hours of incubation at 30°C. (TIF) [file pgen.1005843.s005.tif]

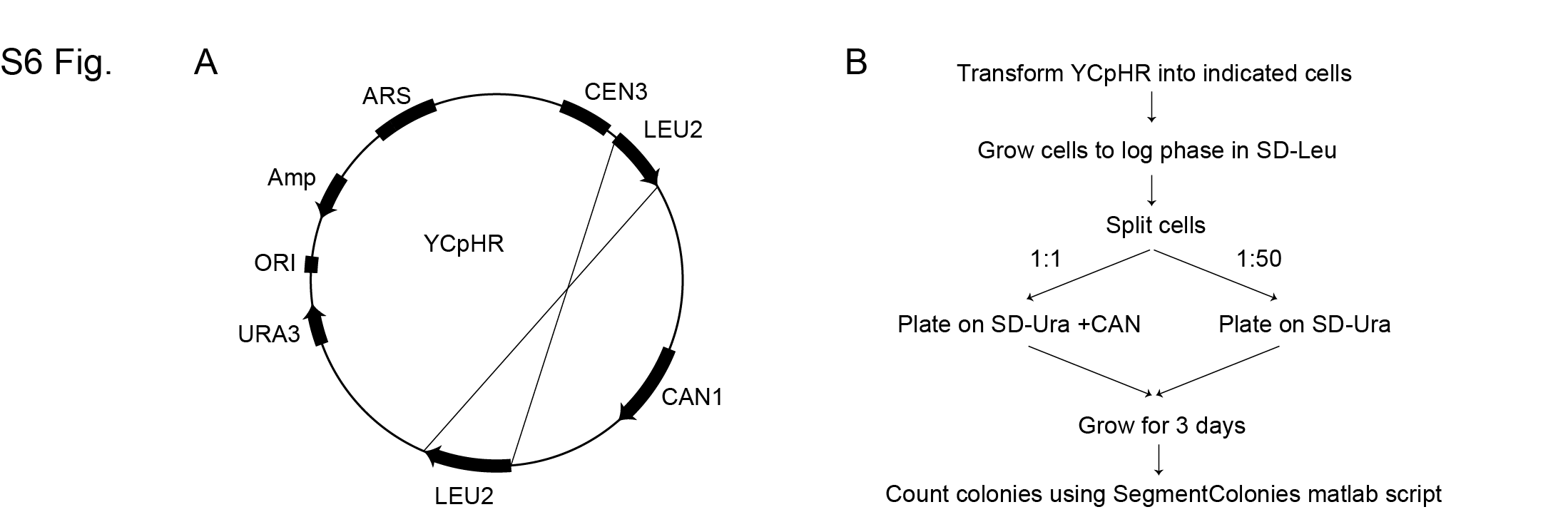

Supplement: S6 Fig — (A) Schematic representation of the YCpHR plasmid reporter [41] used in Fig 5A. (B) Cells transformed with the YCpHR reporter were grown for 5 hours in normal growth conditions (SD-Leu) and plated on either SD-Leu or SD–Leu + canavanine (CAN) media to assess the recombination frequency. CAN resistant colonies were quantified after 72 hours using a SegmentColonies Matlab script. (TIF) [file pgen.1005843.s006.tif]

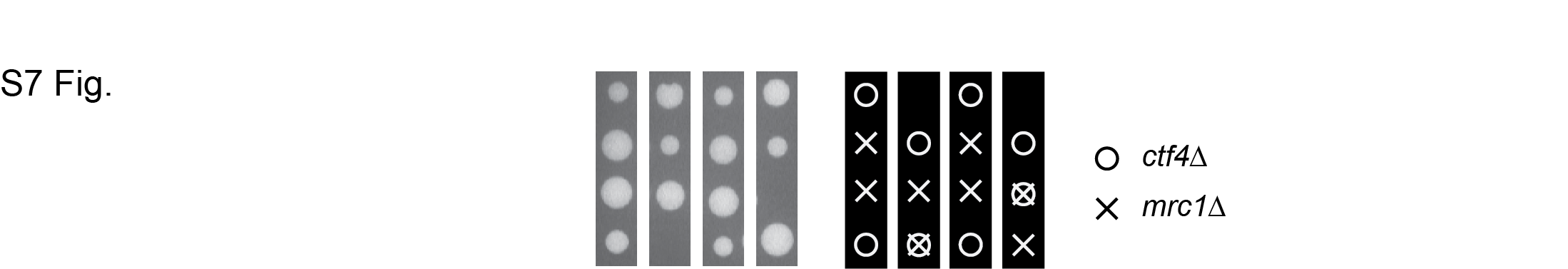

Supplement: S7 Fig — Tetrad analysis from sporulated heterozygote ctf4Δ mrc1Δ diploids. Crosses (X) and circles (O) indicate haploid cells lacking MRC1 or CTF4 respectively. (TIF) [file pgen.1005843.s007.tif]

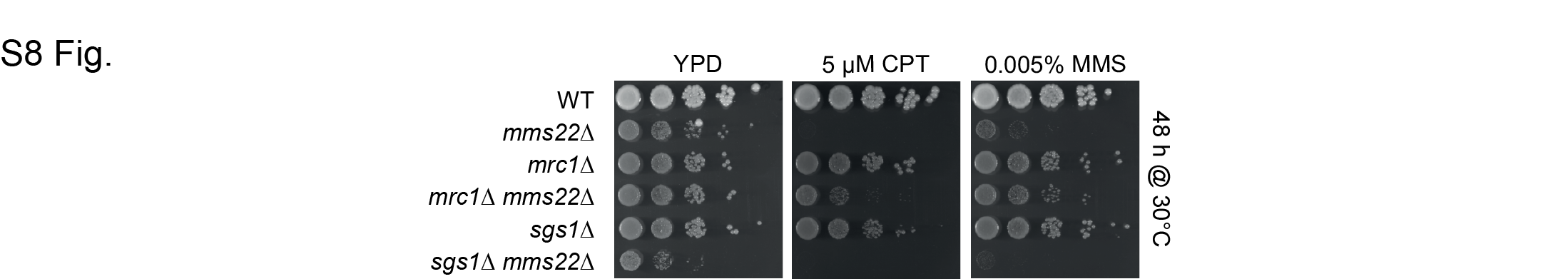

Supplement: S8 Fig — Serial dilution of wild-type (WT) or mms22Δ, mrc1Δ, mms22Δ mrc1Δ, sgs1Δ, mms22Δ sgs1Δ mutants were analyzed on normal growth media (YPD) with or without 0.005% MMS or 5 μM CPT. The plates were imaged after 48 hours of incubation at 30°C. (TIF) [file pgen.1005843.s008.tif]

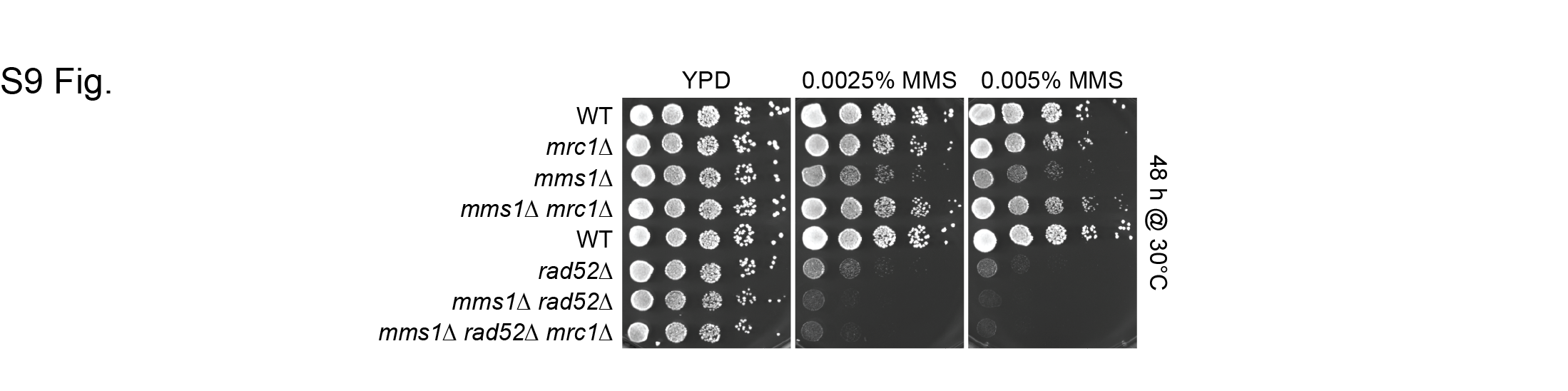

Supplement: S9 Fig — Serial dilution of wild-type (WT) or mrc1Δ, mms1Δ, mms1Δ mrc1Δ and mms1Δ mrc1Δ rad52Δ mutants were assayed on normal growth media and media containing 0.0025% or 0.005% MMS. The plates were imaged after 48 hours of incubation at 30°C. (TIF) [file pgen.1005843.s009.tif]

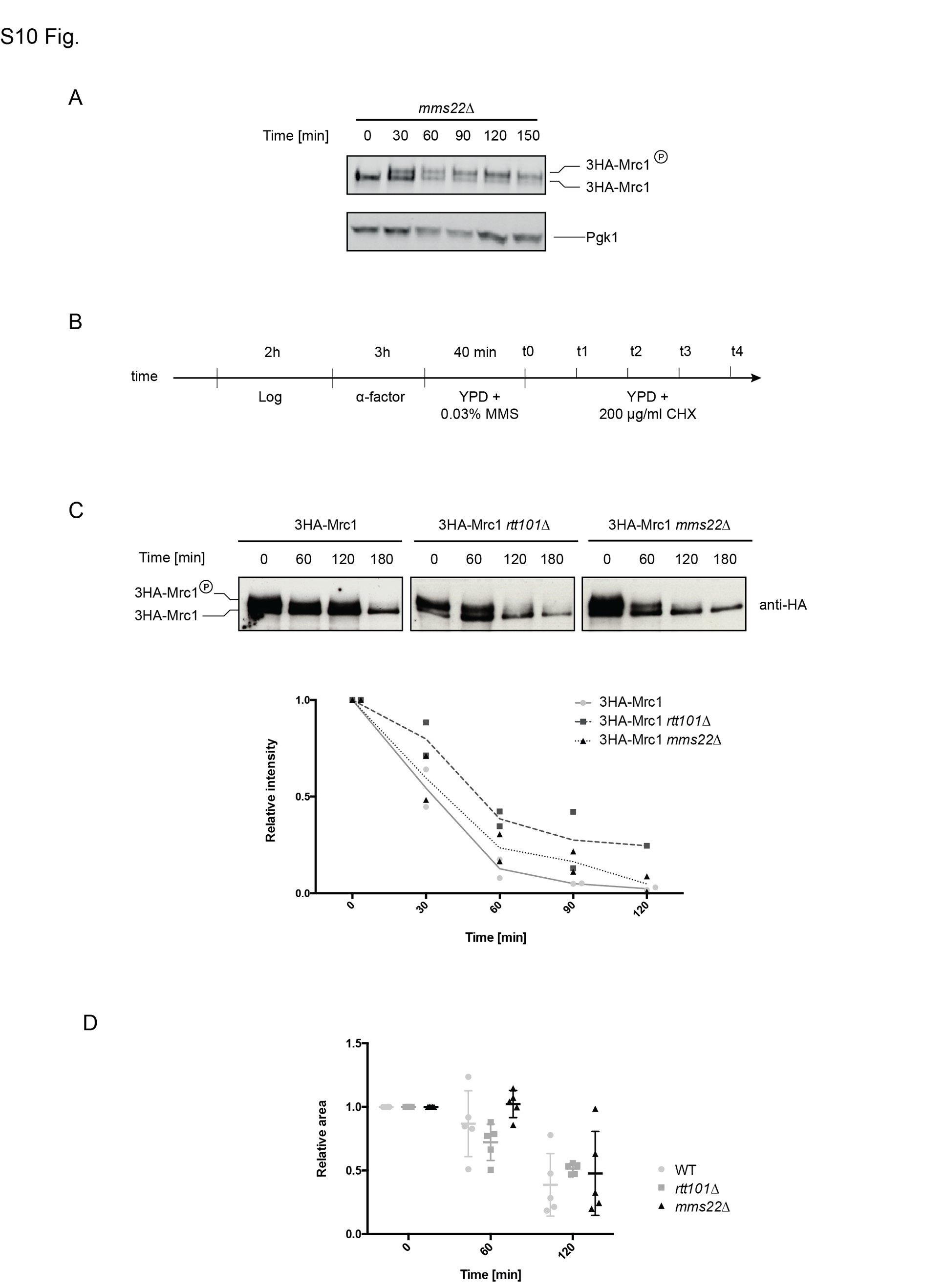

Supplement: S10 Fig — mms22Δ cells expressing 3HA-tagged Mrc1 from the inducible GAL1,10-promoter were synchronized in G1 phase using α-factor in 2% galactose and released into S-phase in 2% galactose as outlined in Fig 6A. Subsequently, 0.03% MMS and 2% glucose was added to induce fork stalling and repress HA-Mrc1 expression, respectively. Samples were collected at the indicated time points (min) and HA-Mrc1 levels monitored by anti-HA immunoblotting (A). Immunoblotting for Pgk1 controls for equal loading. The position of phosphorylated (3HA-Mrc1-P) and unphosphorylated 3HA-Mrc1 is indicated. In an independent approach, wild-type (WT), mms22Δ and rtt101Δ cells expressing 3HA-tagged Mrc1 were synchronized in G1 phase using α-factor in YPD and released into S-phase in YPD + 0.03% MMS as outlined in (B). After 40 min, cells were released in normal growth media containing 200 μg/ml cycloheximide (CHX) and 3HA-Mrc1 was detected at the indicated times by immunoblotting with HA-antibodies (C). The position of phosphorylated (3HA-Mrc1-P) and non-modified (3HA-Mrc1) is marked. Mrc1 protein levels were quantified and normalized from two independent experiments. In addition, endogenous, untagged Mrc1 levels in wild-type (WT), rtt101Δ and mms22Δ were independently quantified by selective-reaction-monitoring (SRM) by measuring transitions corresponding to 5 independent Mrc1 peptides (D). Relative intensities are indicated with standard deviations from five independent peptide measurements. Note that Mrc1 is degraded after release from genotoxic stress by a Rtt101Mms22-independent mechanism. (TIF) [file pgen.1005843.s010.tif]

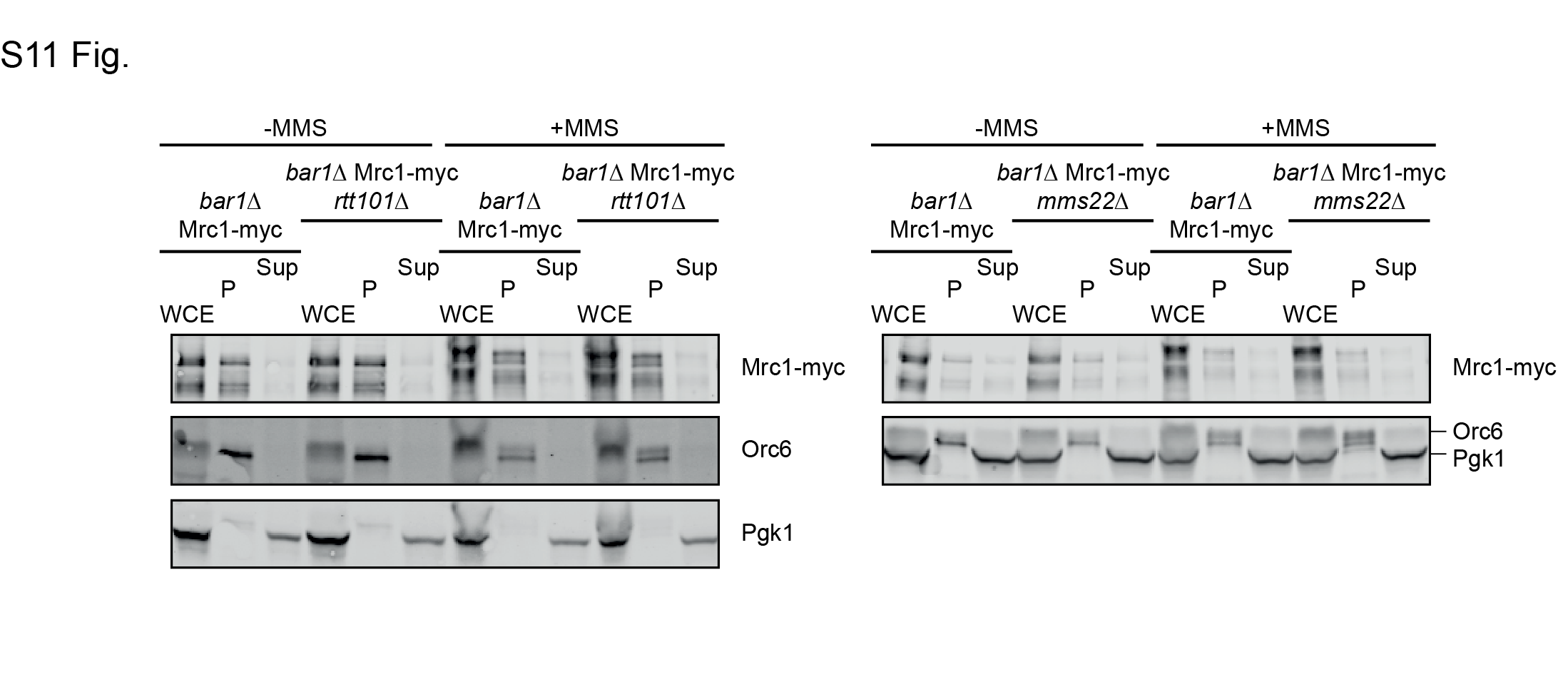

Supplement: S11 Fig — Mrc1-myc expressing strains were synchronized in G1 phase using α-factor and released into medium with (+) or without (-) 0.03% MMS. S-phase samples were collected and the chromatin-bound proteins were separated from the soluble fraction (for detailed experimental procedure see Materials and Methods section). The presence of Mrc1-myc as well as chromatin-associated Orc6 and the soluble Pgk1 controls were detected by immunoblotting in whole cell extract (WCE), the chromatin-associated fraction (pellet = P) and the soluble fraction (supernatant = Sup). (TIF) [file pgen.1005843.s011.tif]
